# Is this the right normalization? A diagnostic tool for ChIP-seq normalization

**DOI:** 10.1186/s12859-015-0579-z

**Published:** 2015-05-09

**Authors:** Claudia Angelini, Ruth Heller, Rita Volkinshtein, Daniel Yekutieli

**Affiliations:** 1 Istituto per le Applicazioni del Calcolo “Mauro Picone”, Via Pietro Castellino, 111, Naples, 80131 Italy; 2 Department of Statistics and Operations Research Tel Aviv University, Ramat Aviv, Tel Aviv, 69978 Israel

**Keywords:** Chip-Seq, Diagnostic plots, Normalization

## Abstract

**Background:**

Chip-seq experiments are becoming a standard approach for genome-wide profiling protein-DNA interactions, such as detecting transcription factor binding sites, histone modification marks and RNA Polymerase II occupancy. However, when comparing a ChIP sample versus a control sample, such as Input DNA, normalization procedures have to be applied in order to remove experimental source of biases. Despite the substantial impact that the choice of the normalization method can have on the results of a ChIP-seq data analysis, their assessment is not fully explored in the literature. In particular, there are no diagnostic tools that show whether the applied normalization is indeed appropriate for the data being analyzed.

**Results:**

In this work we propose a novel diagnostic tool to examine the appropriateness of the estimated normalization procedure. By plotting the empirical densities of log relative risks in bins of equal read count, along with the estimated normalization constant, after logarithmic transformation, the researcher is able to assess the appropriateness of the estimated normalization constant. We use the diagnostic plot to evaluate the appropriateness of the estimates obtained by CisGenome, NCIS and CCAT on several real data examples. Moreover, we show the impact that the choice of the normalization constant can have on standard tools for peak calling such as MACS or SICER. Finally, we propose a novel procedure for controlling the FDR using sample swapping. This procedure makes use of the estimated normalization constant in order to gain power over the naive choice of constant (used in MACS and SICER), which is the ratio of the total number of reads in the ChIP and Input samples.

**Conclusions:**

Linear normalization approaches aim to estimate a scale factor, *r*, to adjust for different sequencing depths when comparing ChIP versus Input samples. The estimated scaling factor can easily be incorporated in many peak caller algorithms to improve the accuracy of the peak identification. The diagnostic plot proposed in this paper can be used to assess how adequate ChIP/Input normalization constants are, and thus it allows the user to choose the most adequate estimate for the analysis.

**Electronic supplementary material:**

The online version of this article (doi:10.1186/s12859-015-0579-z) contains supplementary material, which is available to authorized users.

## Background

Mammalian genomes are organized to form a tridimensional structure called chromatin. It is a highly structured and compact DNA-protein complex that can assume many different conformations depending on the nuclear context and on the biochemical modifications present on both DNA and histone proteins [1]. Its conformation can influence cell activity, state and functionality and can help in understanding why different types of cells exhibit very different behaviours, although they share the same genome. Indeed, transcription factors and chromatin modifiers represent important players in gene regulation, DNA replication, programming and reprogramming of cellular states during differentiation and development. Epigenetic regulatory mechanisms are crucial in the onset and progression of several diseases, including cancer development [2,3], and can be altered by the environment. Therefore, being able to precisely profile all epigenetic actors can deeply help in revealing the landscape of transcription and regulation in cells.

Since the seminal papers [4-7], chromatin immunoprecipitation followed by massively parallel sequencing (ChIP-seq, see [8-10] for a review) has substituted the use of DNA hybridization to microarray (ChIP-chip) and has become a standard technique for identifying protein-DNA interaction [11]. Several large scale projects such as the Encyclopedia of DNA Elements [12,13], The Cancer Genome Atlas (http://cancergenome.nih.gov/), the Roadmap Epigenomics (http://www.roadmapepigenomics.org), are now producing thousands of genome-wide maps for a variety of transcription factors and histone modifications in a large number of different cell types and conditions, see for example [14,15]. In ChIP-seq experiments [8-10], the DNA-binding protein is cross-linked to DNA in vivo, then DNA fragments (usually 200-600 bp) are enriched by immuno-precipitation using an antibody specific to the protein of interest. DNA libraries (i.e. the collections of DNA fragments that will be sequenced) are prepared according to the protocol of the instrument, and finally massively sequenced to produce several millions of short reads of about 50-100 bp.

According to most of the available computational protocols [9,16-18] short reads have to be first aligned to the reference genome in order to identify the genomic locations (i.e., sub-intervals of the whole genome) with enrichment of reads (i.e., with a higher number of mapped reads than expected by chance). Several tools have been proposed for identifying peaks of enriched regions. The corresponding algorithms are known as “*peak caller*” algorithms [19-28]. Different shapes of the genome profile require different peak callers: transcription factors can be well described as sharp punctuate signals of few tens or at most few hundreds base-pairs of length; histone modifications appear as wide-spread and less defined domains and can reach several hundred of kilobases of length; RNA polymerase II binding sites are modelled like a sharp peak with a long heavy tail toward the direction of the transcription.

Exploring the genome-wide coverage profiles it is easy to observe that, due to several experimental sources of errors, the signal consists of both truly enriched regions (i.e., the true regions of interest) and a large number of non specific/non uniform regions that behave as “background”. Following this observation, in [20,29], the data are modeled as a mixture of reads randomly sampled from an enriched signal distribution and a background noise distribution. In order to account for different sources of experimental biases in the background distribution, it is standard practice to sequence a control sample either from Input DNA (i.e., DNA isolated from cells that have been cross-linked and fragmented under conditions similar to those of the experimental sample) or by using a non specific antibody during the enrichment (i.e., IgG) [11]. When the control sample is available, peaks are defined as those sub-regions of the genome with statistically significant higher number of reads than the control/background.

A good peak detection algorithm, that balances sensitivity and specificity, is obtained by choosing an appropriate peak-calling algorithm and a normalization method. The normalization is essential to make the ChIP sample and control/Input sample comparable, since they are usually sequenced with a different number of short reads. The most commonly used procedure for accounting for the different depth is to scale-up the read counts (usually observed in a window *w*) with respect to the total library sizes ratio. This approach is too naive, since it does not account for the fact that only the background component of the ChIP signal follows the same distribution as that of the control signal. Therefore, more sophisticated linear and non-linear approaches to ChIP-seq data normalization have been proposed (see Box 6 and Table S2 in [18] for a summary, and [29-32] for examples). However, despite the substantial impact that the choice of the normalization method can have on the results of a ChIP-seq data analysis [20,29], their properties are not fully explored in the literature, and there are no diagnostic tools that show whether the applied normalization is indeed appropriate for the data being analyzed.

In this paper we focus on linear normalization approaches. These approaches estimate a scale factor, *r*, able to adjust for different sequencing depths. Linear approaches have the great advantage that they can easily be incorporated in many peak caller algorithms to improve the accuracy of the peak identification (e.g., [29]). Moreover, we observe that, independently from the impact on peak calling, the direct estimate of the normalization factor is of interest both for measuring the specificity of the antibody and for comparing specific genomic regions under different conditions. The latter often occurs when relating epigenomic signatures with gene expression, see [33,34].

In this work we propose a novel diagnostic plot to examine the appropriateness of the estimated normalization constant. By plotting empirical densities of log relative risks in bins of equal read count, along with the normalization constant, after logarithmic transformation, the researcher is able to assess the level of agreement of the estimated normalization constant with the data. If the agreement is not satisfactory, the user can revise the estimate, either by using a different normalization method or by changing the input parameters of the method, and reassess. We stress that an accurate diagnosis of the normalization constant is important since it can affect the subsequent analysis dramatically: if the estimated normalization constant is too large, there may be too few discoveries due to power loss in the peak caller algorithms; if the estimated normalization constant is too small, there may be an increase in false positives in the peak caller algorithm.

The paper is organized as follows. After introducing the notation, and three common normalization methods along with their potential limitations, we introduce our diagnostic tool. We examine its usefulness in data-driven simulations based on yeast, as well as on several real mouse data examples and a real model organism example. In order to assess the importance of the correct normalization factor in inference, we examine empirically the effect of different values for the normalization factor on peak calling algorithms, and we examine theoretically the effect of the normalization constant value on the false discovery rate (FDR). We conclude with a summary, some final remarks and future extensions.

## Methods

### Notation

Let *L* be the length of the genome for which the interest is in identifying the locations of a given epigenetic factor or modification. To that purpose, ChIP-seq data for both ChIP and control/Input samples have been produced, and the reads have been uniquely aligned to the reference genome of interest. Let *N*_*ch*_ and *N*_*in*_ denote the number of reads aligned for the ChIP and the Input sample, respectively. The total number of mapped reads is *N*_*tot*_=*N*_*ch*_+*N*_*in*_. Let *π*_0_ be the (unobserved) fraction of reads in the ChIP sample that are background reads. The specificity of the antibody is larger the smaller the value of *π*_0_. Let *r* be the (unobserved) ratio of background reads in the ChIP versus control/Input sample:

(1) $$  r= \frac{\pi_{0} N_{ch}}{N_{in}}  $$

The value of *r* represents the normalization constant of interest. Since *π*_0_ is unknown, it has to be estimated. Note that the naive approach that normalises the reads according to the ratio of the two depths corresponds to setting *π*_0_ to one, which is the correct value only if all the observed reads in the ChIP sample are background reads.

We partition of the genome of interest into *G*_*b*_ windows of equal genomic length |*w*|=*b*, indexed by *w*=1⋯*G*_*b*_. We denote by $\mathcal {W} = \{ 1 \cdots G_{b} \}$ the set of indices of all windows. Let *N*_*ch*_(*w*) be the number of ChIP reads mapped to window *w* and *N*_*in*_(*w*) be the number of Input reads mapped to window *w*. Let *N*_*tot*_(*w*)=*N*_*ch*_(*w*)+*N*_*in*_(*w*) be the total read count in window *w*. The total number of reads in the ChIP and Input samples are therefore $N_{\textit {ch}} = \sum _{w \in \mathcal {W}} N_{\textit {ch}} (w)$ and $N_{\textit {in}} = \sum _{w \in \mathcal {W}} N_{\textit {in}} (w)$, respectively. Remark 1 below discusses the preprocessing steps for the read counts to avoid redundancy in the window counting.

We assume that each enriched region is a compact interval and only a subset of the genome is affected by the modification. These regions may be very large, as expected for histone modifications. The set of windows  is thus comprised of two subsets of indices of enriched and background windows, denoted by $\mathcal {W}_{1} $ and $\mathcal {W}_{0} $, respectively. A window *w* is in $\mathcal {W}_{1} $ if the ChIP signal is enriched, and in $\mathcal {W}_{0} $ if the ChIP signal behaves like background (i.e., control/Input signal).

#### Remark 1.

Since the DNA fragments undergo a PCR step (during the sample preparation) that can result in excessive and non-uniform amplification of the original sequences, it is common practice to remove such artefacts after the alignment step, retaining at most few reads per starting position. When the sequencing depth is low, then the number of retained reads is often only one. In case of higher coverage two or three reads are usually retained. Moreover, to account for the fact that each sequenced read constitutes only the 5’-end of the corresponding DNA fragments, it can be beneficial to shift each mapped position towards their 3’-end by half of the average DNA fragment length before computing *N*_*ch*_(*w*) and *N*_*in*_(*w*).

### Normalization approaches

The unknown ratio *r* in (1) is approximately equal to $\frac {\sum _{w \in \mathcal {W}_{0}} N_{\textit {ch}} (w) } { \sum _{w \in \mathcal {W}_{0}} N_{\textit {in}} (w)}$. The key to estimating *r* is thus the estimation of the subset $\mathcal {W}_{0}$ of background windows. Several normalization procedures have been proposed to estimate *r*, and we shall review the three methods investigated in this paper [20,21,29].

#### CisGenome

In [21], the genome is first divided into windows of length *b*=100 *b* *p* and $\mathcal {W}_{0}$ is estimated by $\hat {\mathcal {W}}_{0}=\left \{ w:\, N_{\textit {tot}} (w)\leq t\right \} $, with *t* fixed at the value *t*=1. The assumption in [21] is that windows with low total count, *N*_*tot*_(*w*), are more likely to belong to the background. The main drawback of this method is that the fixed window-size does not adapt to signals with variable spread, and that the fixed threshold *t*=1 does not scale up with the increase of sequencing depth.

CisGenome is freely available at http://www.biostat.jhsph.edu/~hji/cisgenome/.

#### NCIS

The approach of [29], called NCIS, extends that of [21] by estimating *r* using a data-adaptive length of the window |*w*| and a data-adaptive threshold *t*. The first step is to define ${\hat {\mathcal {W}}_{0}}^{b,t}=\left \{ w: |w|=b ~ ~ \text {and} ~ ~ N_{\textit {tot}} (w) \leq t\right \} $, and compute

$$\hat {r}^{b,t}: = \frac{\sum_{w \in {\hat{\mathcal{W}}_{0}}^{b,t}} N_{ch} (w) } { \sum_{w \in {\hat{\mathcal{W}}_{0}}^{b,t}} N_{in} (w)} $$

for every window size *b* ∈{100 bp;200 bp;500 bp;1000 bp;2000 bp;5000 bp;10000 bp;20000 bp} and for total threshold *t*, where the *t* values considered are all the possible values of total window counts. The second step is to compute for each *b*, $\hat {r}^{b}=\hat {r}^{b,t^{*}} $, where $t^{*}=\min \left \{ t:, \hat {r}^{b,t} \geq \hat {r}^{b,t-1}, ~~ |{\hat {\mathcal {W}}_{0}}^{b,t} | \geq f*G_{b} \right \} $, where *f*∈{0.5,0.75} is an input parameter.

The final step is to estimate ${\mathcal {W}}_{0}$ by ${\hat {\mathcal {W}}_{0}}={\hat {\mathcal {W}}_{0}}^{b^{*},t^{*}}$, where *b* ^∗^ is the smallest window size with an estimated normalization factor that is a local minima (i.e. with value smaller than that computed from smaller window sizes, and at most as large as that of the next largest window size).

This method, as the previous one, assumes that the background windows tend to have lower counts than windows in enriched regions, and therefore may result in poor estimates if the total number of many background windows is actually larger than for windows in enriched regions.

NCIS is an R package available in Additional file two of [29].

#### CCAT

Recognising that due to the intrinsic bias of ChIP-seq experiments, the read counts may also be small for some ChIP-enriched regions and may be relatively large for some background regions, in [20] a different approach has been taken: an iterative method based on the assumption that reads with both positive and negative strand direction are equally distributed in the sample.

The first step is to partition the whole genome into non-overlapping windows of length *b*=1*k* *b* and set $\hat {r}^{0}=\frac {N_{\textit {ch}} }{ N_{\textit {in}} }$. Next, two steps are iterated until convergence:

1. Estimation of ${\hat {\mathcal {W}}_{0}}^{j} $ as follows:
  $${\hat{\mathcal{W}}_{0}}^{j} =\left\{ w:\, N^{+}_{ch}\left(w\right)<\hat{r}^{j-1} N^{+}_{in}\left(w\right)\right\}, $$
  where $N^{+}_{\textit {ch}}(w) $ and $N^{+}_{\textit {in}}(w)$ are, respectively, the ChIP sample and Input sample reads that map on the positive strand only for window w.
2. Updating of $\hat {r}^{j}$ as
  $$\hat {r}^{j}: = \frac{\sum_{w \in {\hat {\cal W}_{0}}^{j} } N^{-}_{ch} (w) } { \sum_{w \in {\hat {\cal W}_{0}}^{j} } N^{-}_{in} (w)}, $$
  where $N^{-}_{\textit {ch}}(w)$ and $N^{-}_{\textit {in}}(w)$ are, respectively, the ChIP sample and Input sample reads that map on the negative strand only for window w.

The estimator may be biased if the distribution of reads in the positive and negative directions differ in many regions.

CCAT is freely available at http://cmb.gis.a-star.edu.sg/ChIPSeq/paperCCAT.htm.

### The diagnostic plot

Given an estimate of *r*, we shall describe the diagnostic plot we propose in order to verify that the estimate of *r* is adequate. Our plot is relevant for bin counts that have a Poisson distribution or a distribution that is more dispersed than the Poisson distribution (e.g., Negative binomial, among others). We shall start by describing our diagnostic tool for bin counts that have a Poisson distribution, then relax this assumption and argue that the diagnostic plot remains relevant for distributions that are more dispersed than the Poisson distribution.

We set an integer *K* in the range from 100 to 1000. For the fixed value of *K*, we partition the genome into (non-equal length) non-overlapping genomic regions, called bins, by agglomerating consecutive windows *w* such that the sum of *N*_*tot*_(*w*) within the bin is (approximately) *K*: the first bin is $\sum _{w=1}^{i_{1}} N_{\textit {tot}}(w)$, where *i*_1_ is the value that satisfies $\sum _{w=1}^{i_{1}-1} N_{\textit {tot}}(w)<K\leq \sum _{w=1}^{i_{1}} N_{\textit {tot}}(w)$; the second bin is $\sum _{w=i_{1}+1}^{i_{2}} N_{\textit {tot}}(w)$, where *i*_2_ is the value that satisfies $\sum _{w=i_{1}+1}^{i_{2}-1} N_{\textit {tot}}(w)<K\leq \sum _{w=i_{1}+1}^{i_{2}} N_{\textit {tot}}(w)$; etc. Hence, a bin aggregates a small number of windows in regions containing many mapped reads in total (i.e., of ChIP plus Input data), and a large number of windows when the total number of mapped reads in the region is low.

For the *i*th bin let $\tilde {N}_{\textit {ch}} (i)$ and $\tilde {N}_{\textit {in}} (i)$ denote the number of reads in the ChIP and Input sample, respectively, and let $\tilde {N}_{\textit {tot}} (i)$ be their total. Let ${\mathcal {B}}_{0}$ denote the set of background bins that contain only windows from ${\mathcal {W}}_{0}$. For bin $i \in {\mathcal {B}}_{0}$, let us first assume that the distribution of $\tilde {N}_{\textit {ch}} (i)$ is Poisson with rate *π*_0_ *N*_*ch*_ *p*(*i*), and the distribution of $\tilde {N}_{\textit {in}} (i)$ is Poisson with rate *N*_*in*_ *p*(*i*), where *p*(*i*) denotes the probability of a read falling in background bin *i*. Therefore, the conditional distribution of $\tilde {N}_{\textit {ch}} (i)$ given $\tilde {N}_{\textit {tot}}(i)$ is Binomial with $\tilde {N}_{\textit {tot}}(i)$ trials and success probability

$$\frac{\pi_{0}N_{ch}p(i)}{\pi_{0}N_{ch}p(i)+N_{in}p(i)} = \frac{r}{r+1}. $$

Let $\pi = \frac {r}{r+1}$ and $\hat \pi = \frac {\tilde {N}_{\textit {ch}} (i)}{\tilde {N}_{\textit {tot}} (i)}$ for *i*∈_0_. For a large enough $\tilde {N}_{\textit {tot}}(i)$, the sample logit, $\log \frac {\hat \pi }{1-\hat \pi }$, has approximate mean $\log \frac {\pi }{1-\pi }$ and standard deviation $1/\sqrt {\tilde {N}_{\textit {tot}}(i) \pi (1-\pi)}$ (see [35] for details). The expressions for the sample logit, its expectation, and its variance simplify as follows:

$$\begin{array}{@{}rcl@{}} \log \frac{\hat \pi}{1-\hat \pi} &=& \log \frac{\tilde{N}_{ch} (i)}{\tilde{N}_{in} (i)}, \quad \log \frac{\pi}{1-\pi}\\ &=& \log r, \quad 1/\sqrt{\tilde{N}_{tot}(i) \pi(1-\pi)}\\ &=& (r+1)/\sqrt{\tilde{N}_{tot}(i)r}. \end{array} $$

The conditional distribution of the log relative risks given $\tilde {N}_{\textit {tot}}(i)$ is approximately normal,

(2) $$ \log \frac{\tilde{N}_{ch} (i)}{\tilde{N}_{in} (i)}\stackrel{\cdot}\sim N\left(\log r,\frac{(r+1)^{2}}{\tilde{N}_{tot}(i)r} \right),  $$

where *N*(*μ*,*σ* ^2^) denotes the normal distribution with mean *μ* and variance *σ* ^2^, and ∼· means “approximately distributed as”. The approximation is better the greater $\tilde {N}_{\textit {tot}}(i)$. Since the bins have approximately the same total counts, it follows that for background bins, the variability of $\log \frac {\tilde {N}_{\textit {ch}} (i)}{\tilde {N}_{\textit {in}} (i)}$ is similar for all *i*, and their expectation is log*r*. Hence, the observed values $\log \frac {\tilde {N}_{\textit {ch}} (i)}{\tilde {N}_{\textit {in}} (i)}$ can be seen as random samples drawn form the same distribution for background bins. A kernel density approach [36] can be used to estimate the density function of $\log \frac {\tilde {N}_{\textit {ch}} (i)}{\tilde {N}_{\textit {in}} (i)}$. For the background bins we expect the peak to be around log*r*. Hence, to assess the performance of the estimate of $\hat {r}$ we can compare the location of $\log \hat {r}$ with respect to the left peak of the estimated density.

**Figure 1:**
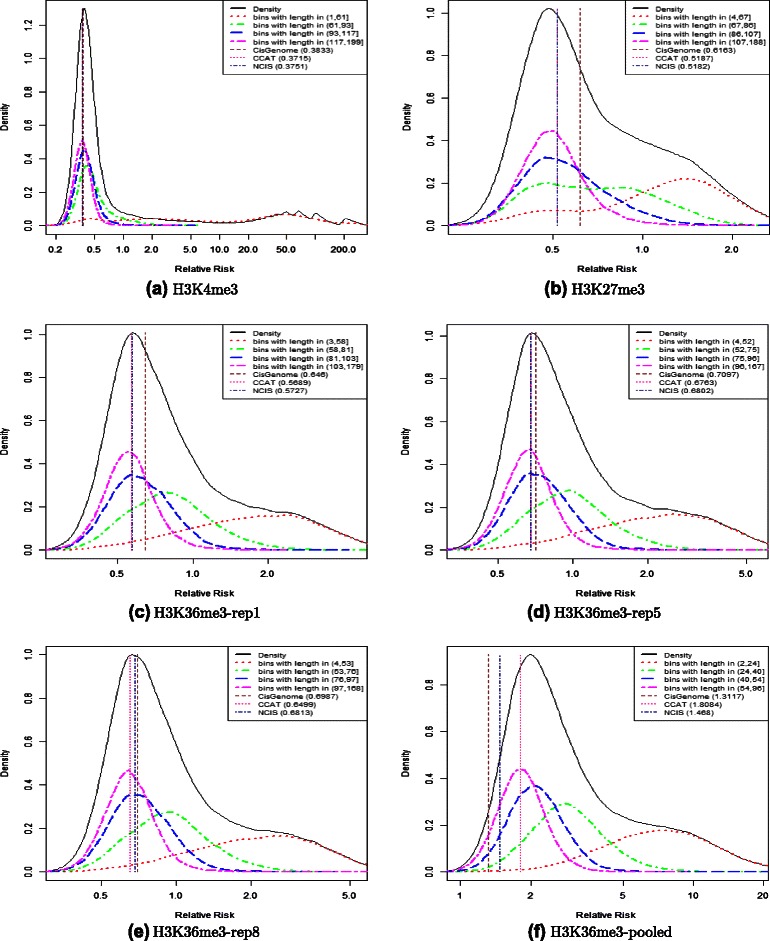
Diagnostic plots for mouse data. Diagnostic plots for six datasets, representing three different modifications, from the mouse embryonic fibroblast cells in the study of [38]. Panel **(a)** refers to H3K4me3, panel **(b)** to H3K27me3, panels **(c-e)** to the three replicates of H3K36me3, finally panel **(f)** to the pooled version of H3K36me3. The five densities are: the density of $\log \frac {\tilde {N}_{\textit {ch}} (i)}{\tilde {N}_{\textit {in}} (i)}$ in all bins (solid black curve), the density of the subset of bins in last quartile in length (two-dashed pink), the density of the subset of bins in third quartile in length (dashed blue), the density of the subset of bins in second quartile in length (dot-dashed green), and the density of the subset of bins in first quartile in length (dotted red). The vertical lines show the estimated log*r* using CisGenome (brown line), CCAT (deepink line) and NCIS (navy line). The plot was produced with *K*=200.

In addition to the estimated density of all the bins, we also plot the four densities of bins in the four quartiles of the bin-length distribution. These additional densities aid in identifying the density for background bins. For example, in the top left Figure 1, which was constructed with *K*=200, the support of the bins ranged from 1 window to 199 windows. The first quartile was 61 windows, the median was 93 windows, and the third quartile was 117 windows. Therefore, in addition to the density of all the bins, we also plot the sub-density for bins that have support of at most 61 windows, the sub-density for bins that have support above 61 windows but at most 93 windows, the sub-density for bins that have support above 93 but at most 117 windows, and the sub-density for bins that have support above 117 windows. Since typically the bins with signal have shorter support, the density of the bins with support in the top quartile of length should describe primarily bins from the background, and the density of the bins with support in the first quartile of length should describe primarily bins that contain signal. Therefore, an adequate estimate of log r should be around the peak of the estimated density of the log relative risks of the bins in the top quartile in length.

**Figure 2:**
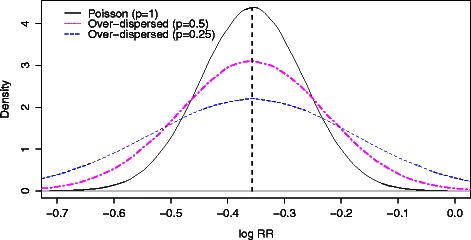
Distributions of estimated log relative risks. The empirical density of the log relative risks for background read counts that are distributed as Poisson (solid black); or as over-dispersed Poisson with *p*=0.5 (dot-dashed pink) and with *p*=0.25 (dash blue). The solid vertical line is *l* *o* *g*(*r*), with *r*=0.7. The peak is around log*r* for all three densities.

In practice the read counts may have a distribution that is more dispersed than the Poisson distribution. We observed that this is so in the data examined in the Results and discussion section, and this has been well documented also by others ([23,25,27], who assumed the distribution of reads was negative binomial). For over-dispersed Poisson read counts, the distribution of $\log \frac {\tilde {N}_{\textit {ch}} (i)}{\tilde {N}_{\textit {in}} (i)}$ is approximately normal with larger standard deviation and slight downward bias. To illustrate this, we performed the following simulation. We set *K*=500, *r*=0.7, and *p*∈{1,0.5,0.25}. We simulated $\tilde {N}_{\textit {ch}}(i)$ and $\tilde {N}_{\textit {in}}(i)$ from a Negative Binomial distribution with rates *r*·*K*/(1+*r*) and *K*/(1+*r*), respectively, and variances $\frac {r \cdot K}{p(1 + r)}$ and $\frac {K}{p(1 + r)}$, and computed the mean and standard deviation of $\log \frac {\tilde {N}_{\textit {ch}} (i)}{\tilde {N}_{\textit {in}} (i)}$. The over dispersion in the simulation is the inverse of *p*, where *p*=1 corresponds to Poisson distribution. The asymptotic mean and standard deviation for the Poisson distribution were *l* *o* *g*(0.7)=−0.3566 and $\sqrt {(1+0.7)^{2}/(500*0.7)}=0.0909.$ In simulations from the two Poisson distributions the mean was −0.3573 and the standard deviation was 0.0911. In simulations from over-dispersed Poisson distributions, the mean and standard deviation were: −0.3580 and 0.1291 for *p*=0.5, i.e. over-dispersion of 2; −0.3596 and 0.1831 for *p*=0.25, i.e. over-dispersion of 4. The simulation standard error was below 0.0001 for all settings. Since the peak of background bins is still supposed to be around log*r* for over-dispersed Poisson, the diagnostic plot remains relevant, see Figure 2.

Finally, we note that the choice of total count, *K*, can matter: if the selected *K* is too small, then the expectation may be farther from log*r* since the large sample approximation is too crude; if the selected *K* is too large then the number of background bins may be too few. For diagnosing whether the actual estimate of log*r* is reasonable, we therefore suggest plotting the empirical density of the log relative risks at several values of *K*. In the Results and discussion section, detailed next, we show that the qualitative assessment of the appropriateness of estimates, in the variety of examples we examined, is the same for different *K* values in the range 100 to 1000. Since our set of examples is representative of enrichment shapes that are encountered in practice, we recommend using the diagnostic plots with *K* in this range.

## Results and discussion

### Data driven simulations

We performed simulations in order to assess the ability of the diagnostic plot to capture the true normalization factor, as well as in pointing out the quality of the estimated normalization factors. We considered two different simulation schemes. They were both based on yeast data, which is 12×10^6^ bp long, about 250 times smaller than that of the human genome.

The first simulation scheme, which we call the “Read-add simulation”, is similar to the simulation method of [29] setting 3. As in [29], it was based on the yeast ChIP-seq study of [37]. The data (GEO Accession number GSE19636) was deeply sequenced: the control sample of segregant 1 (SEG1) had a total of 4.2×10^6^ reads, and the average fragment length was 200 bp. Thus the sequencing coverage was 200×4.2/12=70, much higher than the typical coverage for the human genome. We split the control sample into two halves, “ChIP” and “Input”. We subsampled the two halves to yield 1/*d* of the original library size. Next, we added reads to the “ChIP” half in several locations, as follows. We added at 50 randomly assigned genome locations along the yeast chromosome reads in the range of several thousands base-pairs, with an average increase of 0.2 reads with respect to the “Input” in these enriched regions. In this simulation scheme, the total number of reads was always greater for the “ChIP” library and the true normalization score was 1.

The second simulation scheme, which we call the“By-Genes simulation”, is similar to that of [27]. We downloaded from the Genome Browser the entire table of yeast genome (sacCer1): 27820 partially overlapping gene bodies having lengths ranging from 42 to 632 Kb. The median and average lengths were 1000 bp and 5000 bp, respectively. We selected 300 non-overlapping gene bodies as enriched. We used the control sample SEG1 above, subsampled to yield 1/*d* of the library size in order to estimate the probability of input reads in non-overlapping windows of size 100 bp. We generated the “Input” data from these probabilities. For the “ChIP” data, in enriched regions the probability of reads falling in the windows had a higher probability (about twice as high). In this simulation, the total number of reads is the same for the “ChIP” and “Input” libraries and the true normalization score was determined by the fraction of input reads that fell outside the 300 enriched regions. Specifically, let *p*_*in*_(1),…,*p*_*in*_(*G*) be the fraction of reads in windows 1 to *G* in the 1/*d* sub-sampled control sample SEG1. Then the “Input” number of reads per window was sampled from a multinomial distribution with *N* total reads, and the vector of probabilities *p*_*in*_(1),…,*p*_*in*_(*G*). Let $f_{0} = \sum _{w\in \mathcal {W}_{0}} p_{\textit {in}}(w)$ be the probability of falling in our defined $\mathcal {W}_{0}$ (i.e., the fraction of reads outside the 300 enriched regions out of all the reads in the 1/*d* sub-sampled control sample SEG1). The “ChIP” counts per window were sampled from a multinomial distribution with *N* total reads, and the vector of probabilities *p*_*ch*_(1),…,*p*_*ch*_(*G*), where *p*_*ch*_(*w*)=*λ*_*w*_ *p*_*in*_(*w*)/(*f*_0_+2(1−*f*_0_)), where *λ*_*w*_=2 if $w\in \mathcal {W}_{1}$, and *λ*_*w*_=1 otherwise. Therefore, *r*=*f*_0_/[*f*_0_+2(1−*f*_0_)]∗[1/*f*_0_]=1/(2−*f*_0_).

**Figure 3:**
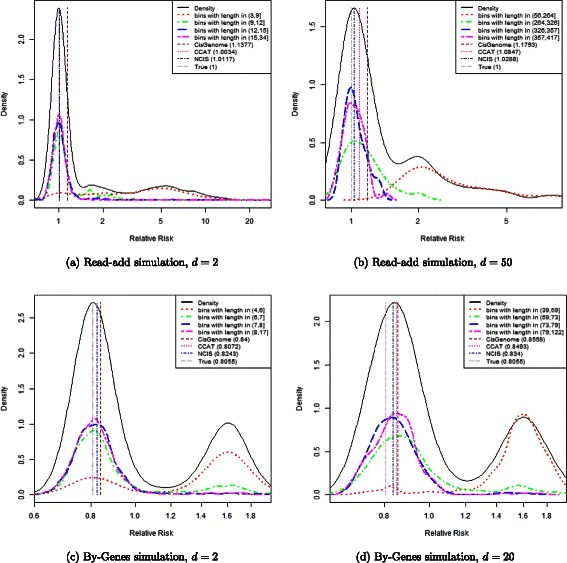
Diagnostic plots for simulated data. Diagnostic plots for four simulated datasets, generated from the control sample of the ChIP-seq study by [37]. Figures **(a)** and **(b)** are the results from the Read-add simulation, with down-sampling by 2 and 50, respectively. Figures **(c)** and **(d)** are the results from the By-Genes simulation, with down-sampling by 2 and 20. The five densities are: the density of $\log \frac {\tilde {N}_{\textit {ch}} (i)}{\tilde {N}_{\textit {in}} (i)}$ in all bins (solid black curve), the density of the subset of bins in last quartile in length (two-dashed pink), the density of the subset of bins in third quartile in length (dashed blue), the density of the subset of bins in second quartile in length (dot-dashed green), and the density of the subset of bins in first quartile in length (dotted red). The vertical lines show the estimated log*r* using CisGenome (brown line), CCAT (deepink line) and NCIS (navy line), as well as the true normalization factor in gray. The plot was produced with *K*=500.

Figure 3 shows that in these data driven simulations, the true normalization factor is indeed close to the peak of the empirical density in the top quartile of length. Therefore, the diagnostic plot judges the true normalization constant to be a good value to use in the statistical analysis. Moreover, the diagnostic plots also judges the estimated normalization factor by CisGenome as slightly biased upwards in settings (a) and (d), and extremely biased downwards in setting (c), as is indeed true when comparing the CisGenome values to the true values. Similarly, the diagnostic plots also suggest that there is a slight upward bias of the other estimates in some of the settings.

### Real data application

#### The diagnostic plot on transcription factors and histone modifications

We applied the normalization methods and diagnostic plot on the ChIP-seq data of mouse embryonic fibroblast cells in [38] (GEO accession number GSE36048).

ChIP-seq samples consist of three histone modifications H3K4me3, H3K27me3 and H3K36me3 and one transcription factor, CTCF, along with the Input sample. The histone modifications are interesting in our context since they have different shapes and levels of enrichment, with H3K4me3 being more compact and peaked around the transcription starting sites of active genes, and H3K27me3 and H3K36me3 more wide-spread over the gene bodies of repressed and active genes, respectively. On the other hand, CTCF is an example of the transcription factor that shows a sharper and more punctuated signal. These experiments contain signals with different spatial resolutions, and we shall apply the diagnostic plot for each of these signal types.

The experimental sequence reads were mapped to the mouse genome assembly mm8 using MAQ version 0.7.1, and these alignments were converted to BED format. The experimental data were subject to filtration, in which we retained at most two reads starting on a single genomic position (see Remark 1). Table 1 summarizes the library sizes before and after the filtration step.

**Table 1:** **ChIP-seq and Input library sizes in the mouse embryonic fibroblast cells in the study of [** 38 **] and in the** ***D. melanogaster*** **in the modENCODE 3955 dataset**

**Modification**	**Library size,**	**Library size, “-2”**	**% of library**
	**bp**	**filtration, bp**	**used**
CTCF [38]	15,995,367	14,378,395	90
H3K4me3 [38]	18,466,608	17,047,383	92
H3K27me3 [38]	12,594,070	11,802,201	94
H3K36me3-1 [38]	16,675,805	14,397,781	86
H3K36me3-5 [38]	20,189,813	17,194,818	85
H3K36me3-8 [38]	19,588,306	16,700,104	85
H3K36me3-pooled [38]	56,453,924	48,292,703	86
Input [38]	19,672,479	17,099,127	87
H3K27me3 (modENCODE,id 1820)	13, 273,869	12,946,455	97
Input (modENCODE,id 1815)	13,425,292	13,168,039	98
H3K27me3 (modENCODE,id 1957)	11,997,853	11,807,914	98
Input (modENCODE,id 1961)	15,685,299	15,402,582	98
H3K27me3-pooled(modENCODE, id 1820and 1957)	25,081,783	24,754,369	98
Input-pooled(modENCODE,id 1815 and 1961)	28,827,874	28,570,621	98

Note the pooled samples where obtained by pooling filtered libraries and did not undergo to a further filtration step.

The H3K36me3 modification had three replicates labeled rep 1, rep 5 and rep 8 in Table 1. Sample H3K36me3-pooled was assembled by pooling the three filtered H3K36me3 replicates. All datasets showed a low percentage of duplicated reads, so the impact of the threshold in the filtering step (for PCR artefact removal) was negligible.

We performed the ChIP/Input normalization factor estimation of the data samples using the CisGenome, NCIS and CCAT methods, using the default parameters in their packages. The diagnostic plots in Figure 1 refer to the histone modification samples (H3K4me3, H3K27me3 and H3K36me3) and include the estimated density and the four sub-densities, as well as the estimated log*r* for the three methods. The assessment was done with *K*=200. The diagnostic plots (for the same modifications) with *K*=50,500,5000 are illustrated in Additional files 1, 2 and 3, respectively. Additional file 4 shows the diagnostic plot for sample CTCF (for *K*=50,100,500 and 2000, respectively).

The shape of the estimated density of the log relative risks confirms that the enriched regions of H3K4me3 are easily separated from those from the background. In fact, the estimated density shows a well-defined peak. For the other two modifications (H3K27me3 and H3K36me3), enrichment is less pronounced and also more widespread, and as a consequence the signal is not as easily separated from the background. This is reflected in the fact that the estimated density shows a less defined peak. However, a comparison of the four sub-densities of the different quartiles in length suggests that the bins in the last quartile in length are background bins, and the bins in the first quartile in length contain mainly signal, for all three modifications.

Since the density of the subset of bins that are in the last quartile of length are background bins, the estimated log*r* is reasonable if it is in the vicinity of the peak of this curve. The diagnostic plot in Figure 1 shows that for the six datasets, the estimator of CCAT is reasonable. The estimate of CisGenome is diagnosed to be too large for the datasets H3K27me3 and H3K36me3-rep 1, and too small for the dataset H3K36me3-pooled. The estimate of NCIS is diagnosed to be too small for the dataset H3K36me3-pooled.

The diagnostic plot for CTCF, in Additional file 4, shows that the estimates by CisGenome and NCIS (which are very close) agree best with the data, and that the estimate of CCAT may be slightly too small: the mode of the empirical density of the upper quartile in length bins (in pink) for all values of *K* is very close to the estimates by CisGenome and NCIS, and slightly to the right of the estimate by CCAT.

Table 2 shows the estimated *r* and *π*_0_ for each method in each dataset. Since the dataset H3K36me3-pooled is the pooled data from the three replicates rep 1, rep 5, and rep 8, another estimator for *π*_0_ in the pooled data can be obtained by a weighted average of the estimates in the three replicates:

$$\tilde{\pi}_{0p} = \frac{\hat{\pi}_{01}N_{Ch_{1}}+\hat{\pi}_{05}N_{Ch_{5}}+\hat{\pi}_{08}N_{Ch_{8}}}{N_{Ch_{1}}+N_{Ch_{5}}+N_{Ch_{8}}}, $$

where $\hat \pi _{0i}$ and $N_{Ch_{i}}$ are the estimated fraction of nulls and the total number of reads in the ChIP study in rep *i*∈{1,5,8}. The estimate of *π*_0_ is connected to the specificity of the antibody. When the same batch of antibody is used to enrich different replicated libraries (within analogous laboratory conditions), we expect that the specificity will be similar on different replicates, while significant differences can be viewed as a sign of a different behaviour of the antibody. On the other hand, when pooling different samples, the specificity of the antibody is the linear combination of the specificity within each library weighted by the proportion of each library in the pool. The direct comparison of the estimates of NCIS and CisGenome from the pooled data in row 4 of Table 2 with the estimate $\tilde {\pi }_{0p}$ indicates clearly that their direct estimate is far too small. This concurs with the finding from the diagnostic plot, that the estimates of NCIS and CisGenome are too small for the dataset H3K36me3-pooled, and deviate from the expected value inferred from those obtained using single replicates.

**Table 2:** **For each dataset of [** 38 **], the estimated** ***r*** **and** ***π***_**0**_ **from CisGenome (columns 2 and 3), from NCIS (columns 4 and 5), and from CCAT (columns 6 and 7)**

**Dataset**	**CisGenome**		**NCIS**		**CCAT**	
	$\boldsymbol {\hat {r}}$	$\boldsymbol {\hat {\pi }_{0} = \frac {N_{\textit {in}}}{N_{\textit {ch}}}\hat {r}}$	$\boldsymbol {\hat {r}}$	$\boldsymbol {\hat {\pi }_{0} = \frac {N_{\textit {in}}}{N_{\textit {ch}}}\hat {r}}$	$\boldsymbol {\hat {r}}$	$\boldsymbol {\hat {\pi }_{0} = \frac {N_{\textit {in}}}{N_{\textit {ch}}}\hat {r}}$
CTCF	0.6699	0.5633	0.6657	0.5598	0.6419	0.5397
H3K4me3	0.3833	0.3845	0.3751	0.3762	0.3715	0.3726
H3K27me3	0.6163	0.8929	0.5182	0.7507	0.5187	0.7515
H3K36me3-1	0.6460	0.7672	0.5727	0.6801	0.5689	0.6757
H3K36me3-5	0.7097	0.7058	0.6802	0.676	0.6763	0.6725
H3K36me3-8	0.6987	0.7154	0.6813	0.6976	0.6499	0.6654
H3K36me3-pooled	1.3117	0.4644	1.4680	0.5198	1.8084	0.6403
$\frac {\hat {\pi }_{01}N_{Ch_{1}}+\hat {\pi }_{05}N_{Ch_{5}}+\hat {\pi }_{08}N_{Ch_{8}}}{N_{Ch_{1}}+N_{Ch_{5}}+N_{Ch_{8}}}$		0.7274		0.6848		0.6710

The last row shows an estimate of *π*_0_ for the pooled sample (modification H3K36me3) based on a weighted average of the three estimated *π*_0_ in the three individuals samples.

#### Effect of the coverage depth

It is known that very disperse histone modifications such as H3K27me3 require very high coverage to be identified, and the coverage in the dataset of [38] was low. Therefore, in addition to the examination of H3K27me3 in the data of [38], we examined it in a study on *D. melanogaster* from modENCODE Project[39] (Model Organism ENCyclopedia Of DNA Elements). The dataset consists of two replicates of histone modification H3K27me3 (identified by id number 1820 and 1957) and two replicates of Input (identified by id number 1815 and 1961) from Drosophila Oregon R embryos, 14-16 hr after egg laying (GEO accession number GSE47230, modENCODE 3955).

Short reads were aligned to the reference genome using Bowtie and the alignments were converted to BED format. As before, the aligned dataset was subject to filtration, meaning that up to two reads were retained in starting on a single genomic position.

Table 1 shows the number of reads sequenced in the study. Since the *D. melanogaster* genome is about 1.2∗10^8^ bp (compared to 2.8∗10^9^ bp for the mouse genome), the coverage of this dataset was about 20 times higher than the one in previous examples. Sample H3K27me3-pooled was assembled by pooling the two filtered H3K27me3 replicates. In a similar way we also pooled the two replicate of the Input.

**Figure 4:**
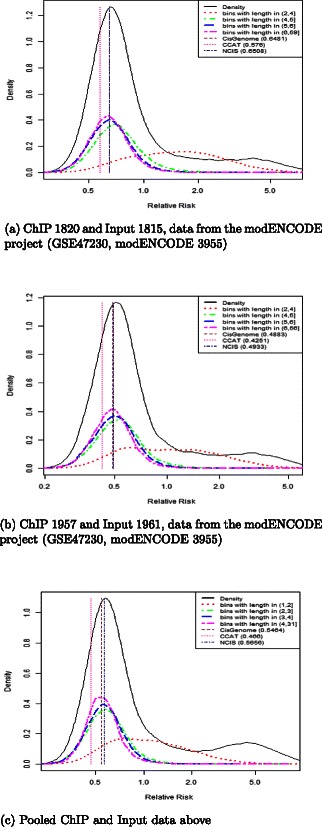
Diagnostic plots for modENCODE data. Diagnostic plots for the three datasets from modENCODE. Datasets refer to H3K27me3 modification in *D melanogaster*. Panel **(a)** refers to ChIP id. 1820 and Input id. 1815, panel **(b)** to ChIP id 1957 and Input id 1961, panel **(c)** to the pooled version of the modEncode semples. The five densities are: the density of $\log \frac {\tilde {N}_{\textit {ch}} (i)}{\tilde {N}_{\textit {in}} (i)}$ in all bins (solid black curve), the density of the subset of bins in last quartile in length (two-dashed pink), the density of the subset of bins in third quartile in length (dashed blue), the density of the subset of bins in second quartile in length (dot-dashed green), and the density of the subset of bins in first quartile in length (dotted red). The vertical lines show the estimated log*r* using CisGenome (brown line), CCAT (deepink line) and NCIS (navy line). The plot was produced with *K*=200.

Table 3 shows the estimated *r* and *π*_0_ for each method in each dataset. The diagnistic plot is shown in Figure 4 and it includes the estimated density and the four sub-densities as well as the estimated log *r* for the three methods. The assessment was done with *K*=200.

**Table 3:** **For each dataset of modENCODE 38955, the estimated** ***r*** **and** ***π***_**0**_ **from CisGenome (columns 2 and 3), from NCIS (columns 4 and 5), and from CCAT (columns 6 and 7)**

**Dataset**	**CisGenome**		**NCIS**		**CCAT**	
	$\boldsymbol {\hat {r}}$	$\boldsymbol {\hat {\pi }_{0} = \frac {N_{\textit {in}}}{N_{\textit {ch}}}\hat {r}}$	$\boldsymbol {\hat {r}}$	$\boldsymbol {\hat {\pi }_{0} = \frac {N_{\textit {in}}}{N_{\textit {ch}}}\hat {r}}$	$\boldsymbol {\hat {r}}$	$\boldsymbol {\hat {\pi }_{0} = \frac {N_{\textit {in}}}{N_{\textit {ch}}}\hat {r}}$
H3K27me3 (modENCODE,id 1820 vs id 1815)	0.6481	0.6587	0.6508	0.6615	0.5760	0.5855
H3K27me3 (modENCODE,id 1957 vs id 1961)	0.4883	0.6364	0.4933	0.6428	0.4251	0.5540
H3K27me3-pooled (modENCODE)	0.5464	0.6301	0.5656	0.6522	0.4660	0.5373
$\frac {\hat {\pi }_{1820}N_{Ch_{1820}}+\hat {\pi }_{1957}N_{Ch_{1957}}}{N_{Ch_{1820}}+N_{Ch_{1957}}}$		0.6473		0.6520		0.5694

The last row shows an estimate of *π*_0_ for the pooled sample based on a weighted average of the estimated *π*_0_ in the two individuals samples.

The shape of the estimated density of the log relative risks suggests that the enriched regions can be well separated from the background. The empirical density of the lower and upper quartile in length (red and pink curves, respectively) clearly capture the enriched and background bins. The diagnostic plot shows that the estimates by CisGenome and NCIS (which are very close), agree best with the data, and that the estimate of CCAT is too small: the mode of the empirical density of the upper quartile in length bins (in pink) is very close to the estimates by CisGenome and NCIS, and it is to the right of the estimate by CCAT. Additional file 5 shows the diagnostic plot for ChIP sample id 1820 versus Inpunt sample Id 1815 and *K*=100,200,1000, and 2000, respectively (the other cases behave similarly and are not shown for brevity).

### Impact on peak calling algoritms

The main purpose of the ChIP-seq studies is to detect binding sites and/or enriched regions of histone modifications, and a peak detection algorithm is typically used for this purpose. In general, the impact of the normalization on the identified regions depends on the specific peak calling method and on the type of multiple testing procedure that is implemented. Most of the peak calling algorithms normalize data inside their code using suitable internal strategies. Tools such as MACS [24] and SICER[25] use *N*_*ch*_/*N*_*in*_ as their normalization constant. Following the suggestion of [29], we modified both MACS and SICER, making them able to work with user defined normalization constants. For both methods, the (global) normalization constant does not change the rank of the peaks (where the ranking is by *p*-values or by enrichment scores), but it does affect the number of discovered regions. Therefore, a smaller normalization constant, with all other parameters held fixed, may result in a larger number of discovered regions.

Table 4 shows the empirical impact of the normalization constant value. The number of peaks identified using the default normalization constant (column 2), was always smaller than the number of peaks identified using the estimated normalization constants by CisGenome, NICS, and CCAT (columns 3, 4, and 5, respectively). By referring to the values of the estimated normalization constants in Tables 2 and 3 we see that the number of discoveries increased as the input constant decreased. From Table 4 we see that the choice of the normalization constant had a significant impact on the peak selection in the datasets H3K36me3-pooled, CTCF, and all the series of modEncode H3K27me3. The impact was less pronounced in the other datasets. The empirical impact of the normalization constant on the peak identifications was shown to be drammatic by [29] for transcription factors.

**Table 4:** **Number of peaks detected using the naive normalization constant (column 2), the estimate by CisGenome (column 3), NCIS (column 4), and CCAT (column 5), in a specific peak calling algorithm (column 6)**

**TF or Modification**	**Default**	**CisGenome**	**NCIS**	**CCAT**	**Peak calling Algorithm**
CTCF [38]	22522	29841	30369	38339	MACS
H3K4me3 [38]	16972	17400	17401	17403	SICER
H3K27me3 [38]	10233	10844	11272	11271	SICER
H3K36me3-1 [38]	10139	10676	10747	10752	SICER
H3K36me3-5 [38]	13880	15192	15248	15257	SICER
H3K36me3-8 [38]	14079	15454	15494	15564	SICER
H3K36me3-pooled [38]	16326	18788	18729	18509	SICER
H3K27me3 (modENCODE:id 1820 vs id 1815)	1037	1959	1955	2028	SICER
H3K27me3 (modENCODE:id 1957 vs id 1961)	1026	1894	1893	1949	SICER
H3K27me3-pooled (modENCODE)	1118	2068	2049	2172	SICER

SICER was applied on histone modifications with the following input values: window size 200, gap size 400, FDR 10^−8^ and all other parameters as default. MACS was applied on CTCF using default values and FDR 10^−4^. The FDR considered here is the one implemented in the corresponding software.

Moreover, by looking at the locations of the enriched regions along the chromosomes in the two replicates we also measured the number of overlapping/non overlapping intervals as function of the normalization constant. First, we considered the 1037 regions detected as enriched (with default constant) in the first H3K27me3 replicate of modEncode dataset, and the 1026 regions detected as enriched in the second replicate (see Table 4, column 2). We found that 106 regions of the first set did not have any positional overlap with the regions in the second set; and 154 regions of the second set did not have any overlap with the the first set. However, out of the 106 regions above described, 79 are detected as enriched in the second replicate when using NCIS as constant (i.e., they overlap some of the 1893 regions in Table 4, column 4), and out of the 154 regions, 113 overlaps those of the first replicate when using NCIS as constant (i.e., the 1955 regions Table 4, column 4). Such comparisons suggest that a proper estimate of the normalization constant can increase the number of true discoveries: for example 79 peaks that were detected by the first replicated but not by the second replicate using the naive constant due to lack of power, but when the proper normalization constant was used they were indeed detected in both replicates.

Overall, using NCIS for estimating the normalization constant, out of the 1955 regions of the first replicate, 1657 overlap the 1893 regions of the second replicate; and out of the 1893 regions of the second replicate 1542 overlap the 1955 regions of the first replicate.

### The effect of the estimated normalization constant on the FDR

As mentioned in the above section, the normalization constant has an impact on the number of regions that are declared significant. Each peak calling algorithm produces an enrichment score (or a p-value) for each region of interest. The greater the enrichment score (the smaller the p-value), the greater the evidence that there is a modification or a binding site in that region. In order to determine the cutoff threshold, above which a region is considered enriched, it has been suggested by [20] and [29] (among others) to use false discovery rate (FDR, [40]) estimation by library swapping.

The estimated normalization constant has a crucial role in determining the cutoff threshold. If the estimate is too large, $\hat r>r$, the procedure may be overly conservative. If the estimate is too small, $\hat r<r$, the procedure may detect too many false positives. To see this, we first introduce a peak detection procedure based on library swapping that controls the FDR, and then we discuss the effect of over- or under- estimation of *r*.

Consider an enrichment score $g(\tilde {N}_{\textit {ch}} (i), \tilde {N}_{\textit {in}} (i), r)$, where $\tilde {N}_{\textit {ch}} (i)$ and $\tilde {N}_{\textit {in}} (i)$ denote the number of reads in the ChIP and Input sample, respectively, in bin *i*. The swapped score in bin *i* is therefore $g(\tilde {N}_{\textit {in}} (i), \tilde {N}_{\textit {ch}} (i), 1/r)$. Let *q* be the desired FDR level (e.g., *q*=0.05). Let $\mathcal {S} = \{i: g(\tilde {N}_{\textit {in}} (i), \tilde {N}_{\textit {ch}} (i), 1/r)\geq g(\tilde {N}_{\textit {ch}} (i), \tilde {N}_{\textit {in}} (i), r)\}$ be the index set of the bins where the enrichment score is higher for the swapped libraries. Let $\mathcal {S}^{c}$ contain the remaining indices.

#### Procedure 1.

The peak detection procedure at FDR level *q* is as follows:

1. Find
  $${\fontsize{7.6pt}{9.6pt}\selectfont{\begin{aligned} T= \min \left\{ t\in (0,\infty): \frac{1+\#\{i\in \mathcal{S}:g(\tilde{N}_{in} (i), \tilde{N}_{ch} (i), 1/r)\geq t \}}{\#\{i\in \mathcal S^{c}:g(\tilde{N}_{ch} (i), \tilde{N}_{in} (i), r)\geq t \}\lor 1}\leq q \right\}\!. \end{aligned}}} $$
2. Declare all bins with $g(\tilde {N}_{\textit {ch}} (i), \tilde {N}_{\textit {in}} (i), r)\geq T$, $i \in \mathcal S^{c}$, as enriched.

#### Theorem 1.

Suppose that the enrichment scores in non-enriched (i.e., null) regions are i.i.d and are independent from the scores in enriched regions. Moreover, suppose that for a non-enriched (i.e., null) bin *i*,

$$ Pr\left(g\left(\tilde{N}_{in} (i), \tilde{N}_{ch} (i), 1/r\right)<g\left(\tilde{N}_{ch} (i), \tilde{N}_{in} (i), r\right)|G_{i}\right)\leq 1/2, $$

where $G_{i} = \max \{g(\tilde {N}_{\textit {ch}} (i), \tilde {N}_{\textit {in}} (i), r),g(\tilde {N}_{\textit {in}} (i), \tilde {N}_{\textit {ch}} (i), 1/r)\}$. Then the FDR of the above procedure is controlled at level *q*.

See Additional file 6 for a proof based on a very nice recent result of [41].

Since *r* is not known in practice, it is estimated from the data. For a reasonable enrichment score, $g(\tilde {N}_{\textit {ch}} (i), \tilde {N}_{\textit {in}} (i), r)$ decreases with increasing *r*, since a larger *r* implies that a larger fraction of the reads in the ChIP sample are null reads. Consider an estimate $\hat r>r$. Then $g(\tilde {N}_{\textit {ch}} (i), \tilde {N}_{\textit {in}} (i), \hat {r})< g(\tilde {N}_{\textit {ch}} (i), \tilde {N}_{\textit {in}} (i), r)$, and $g(\tilde {N}_{\textit {in}} (i), \tilde {N}_{\textit {ch}} (i), 1/\hat {r})>g(\tilde {N}_{\textit {in}} (i), \tilde {N}_{\textit {ch}} (i), 1/ r)$. Therefore, using $\hat {r}$ instead of *r*, may result in a larger set  (and, respectively, a smaller set $\mathcal {S}^{c}$), and the cut-off threshold based on $\hat {r}$ will be smaller, thus less discoveries would be made. A similar reasoning suggests that if $\hat {r}<r$, the cut-off threshold based on $\hat {r}$ will be larger, thus more discoveries would be made. However, if $\hat r<r$, there is no longer any guarantee for FDR control, and it may very well be that the actual fraction of false discoveries among the discoveries is far larger than the desired level *q*. Therefore, in order to keep the desired balance between power and false discovery control, it is crucial to estimate *r* well.

#### Remark 2.

The algorithm does not require a full list of enrichment scores for all bins considered, and a partial list of the largest scores may suffice. To see this, note that the procedure can be executed as follows (the justification follows from the proof in Additional file 6.

1. Sort the bins, so that the bin with index *i*=1 has the largest enrichment score or swapped score, the bin with index *i*=2 has the second largest enrichment score or swapped score, etc. Formally, if $G_{i} = \max \left \{g(\tilde {N}_{\textit {ch}} (i), \tilde {N}_{\textit {in}} (i), r),g(\tilde {N}_{\textit {in}} (i), \tilde {N}_{\textit {ch}} (i), 1/r)\right \}$, the sorted scores satisfy *G*_1_≥…≥*G*_*B*_>0, where *B* is the total number of bins considered for enrichment.
2. Find
  $$\hat k= \max \left\{ k : \frac{1+\#\{i\leq k, i \in \mathcal{S} \}}{\#\{i\leq k, i \in \mathcal{S}^{c} \}\lor 1}\leq q \right\}. $$
3. Declare the bins $i=1,\ldots, \hat {k}$ as enriched.

If a peak calling algorithm outputs only all enrichment scores above a cut-off *A*, then the inference proceeds as follows. The *G*_*i*_s are computed for all the bins with enrichment score or swapped score above *A*. Clearly, if bin *i* was only discovered in the original analysis, then $i\in \mathcal {S}^{c}$, and if it was only discovered after swapping the libraries, then $i\in \mathcal {S}$. If the bin was discovered by both original analysis and analysis after swapping, then the bin is in $\mathcal {S}^{c}$ if the score in the original analysis was highest, and it is in  if the score after library swapping is highest. If $\frac {1+\#\{i \in S \}}{\#\{i \in \mathcal {S}^{c} \}\lor 1}\leq q$, all bins above the cut-off *A* are declared enriched. Otherwise, find $\hat k$ as detailed above, and declare the bins $i=1,\ldots, \hat k$ as enriched. It is straightforward to show that the selection above the cut-off *A* does not invalidate the procedure, and thus the FDR is still controlled. The cut-off *A* may however affect the power, since if a lower cut-off than *A* would have led to more rejections, then the power could have been higher. Therefore, it is desirable to choose a cut-off *A* below which it is believed that enrichment is unlikely.

### Code availability

The diagnostic plot described in this paper has been implemented within the *c* *h* *i* *p*_*d* *i* *a* *g* *n* *o* *s* *t* *i* *c* *s* function in the R language. The *c* *h* *i* *p*_*d* *i* *a* *g* *n* *o* *s* *t* *i* *c* *s* function is described in the Additional file 7.

## Conclusions

The analysis of ChIP-seq data has become in the last decade one of the most used methods for obtaining genome-wide maps of protein-DNA interactions and different epigenetic signatures. Despite the many available tools for detecting enriched regions from ChIP-seq experiments, many statistical issues are still open. Among them, we have focused our attention on the problem of estimating the normalization constant when comparing ChIP and Input samples. We developed a simple diagnostic tool for assessing the appropriateness of a given normalization constant when applied to a real dataset. We illustrated the proposed approach on several real datasets consisting of enriched regions of various shapes. We showed the usefulness of such a plot in picking the most reliable constant among few proposals. As a consequence, instead of choosing one specific estimator for all datasets, the user can choose the estimator that is most adequate for the dataset under analysis.

All our examples clearly show that there is no single normalization method that is clearly superior to the other ones, under different settings. The diagnostic tool can identify problematic cases that require a reassessment of the normalization procedure, and it can choose the best estimate among several. However, it cannot serve as an estimate of the normalization constant, even though the diagnostic tool shows graphically the range of reasonable estimates. This is so since from the graph it is very difficult to extract a single number which can serve as an estimate of the normalization constant. Although the left peak of the empirical density of the log relative risks is expected to be around log*r*, we see from the real and simulated examples that the peak is broad, and that it depends on *K*. Therefore, it is hard to estimate the normalization constant from the diagnostic plot, and such an estimate will necessarily be unstable and of limited use.

Once the normalization constant is chosen, it should be incorporated in the algorithm of detection of enriched regions in peak calling tools. Unfortunately, most of the tools compute the normalization constant directly in their code. Therefore, to change the constant the user has to access the source code. By suitably modifying MACS and SICER, we show that the list of significant regions can be tuned using $\hat {r}$. An over-estimate of $\hat {r}$ will produce an over conservative list of peaks, an under-estimate will increase the number of false positive. We also provide a novel procedure aimed to control the FDR at level *q* using a sample swapping strategy. This novel procedure can be incorporated in the analysis pipeline to allow a more rigorous control of false positives.

In this paper we have considered the problem of between-sample normalization, and we have limited our attention to linear methods (i.e., a global estimator $\hat {r}$). However, the problem of sample normalization in the ChIP-seq context can be more complex than what has been considered here. In fact, several other experimental biases are connected with ChIP-seq normalization, such as CG content, PCR amplification, library preparation etc. This suggests that both within sample and between sample normalization procedures should be applied. Some non-linear methods towards this direction have already been proposed in the context of ChIP-seq [30,31]. Other approaches could be derived from methods available for DNA-seq (see [42] for CG content bias). Our approach does not directly apply to non linear methods and the extension might depend on the type of non liner method that is considered. Such extension is outside the scope of this manuscript, and a direction for future research.

In this paper we have considered the case where a ChIP sample is compared with an Input sample. It is now becoming standard practice to profile more ChIP samples (relative to the same transcription factor or histone modification) collected under different experimental conditions, such as those used to investigate the epigenetic response to different pharmacological treatments or to associate difference in the epigenetic profile to different diseases status. In such cases the problem is to detect regions that are both enriched with respect to the respective Input samples and are differentially enriched among the ChIP samples belonging to different experimental conditions. This context constitutes an emerging area of research with relatively few methods available (see [18] for a review), where normalization is going to play a very important role. Our diagnostic tool is expected to be useful in such settings as well.

Another point of future development is the possibility of incorporating additional source of biological information such as chromatin accessibility obtained from by DNase I digestion [43] in order to create the sub-densities.

## Additional files

Additional file 1
**Diagnostic plots.** Analogous to Figure 1. Diagnostic plots for six datasets of histone modifications in [38]. The plot refers to *K*=50.

Additional file 2
**Diagnostic plots.** Analogous to Figure 1. Diagnostic plots for six datasets of histone modifications in [38]. The plot refers to *K*=500.

Additional file 3
**Diagnostic plots.** Analogous to Figure 1. Diagnostic plots for six datasets of histone modifications in [38]. The plot refers to *K*=5000.

Additional file 4
**Diagnostic plots.** Diagnostic plots for the CTCF dataset in [38]. The plot refers to *K*=50,100,500 and 2000, respectively.

Additional file 5
**Diagnostic plots.** Analogous to Additional file 4. Diagnostic plots for H3K27me3 dataset in modENCODE 3955 (id 1820 vs id 1815). The plot refers to *K*=100,500,1000 and 2000, respectively.

Additional file 6
**Proof of Theorem 1.**

Additional file 7
**R function** ***chip*** **_** ***diagnostics*** **.** The supplementary html file illustrates the *c* *h* *i* *p*_*d* *i* *a* *g* *n* *o* *s* *t* *i* *c* *s* function and its usage.
