# Supplementary figures and images for: Is this the right normalization? A diagnostic tool for ChIP-seq normalization
Source: BMC Bioinformatics. 2015 May 9;16:150. doi: 10.1186/s12859-015-0579-z (PMC4448883; doi:10.1186/s12859-015-0579-z)

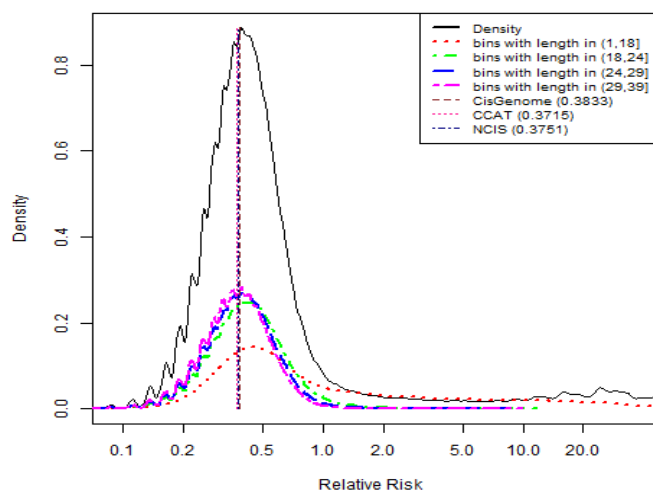

(a) H3K4me3

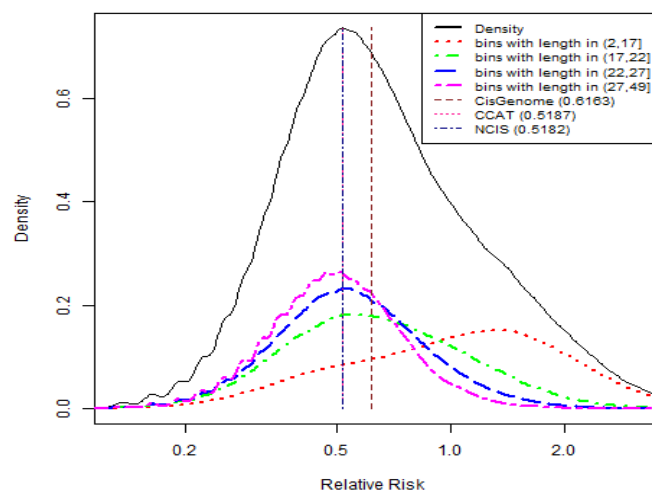

(b) H3K27me3

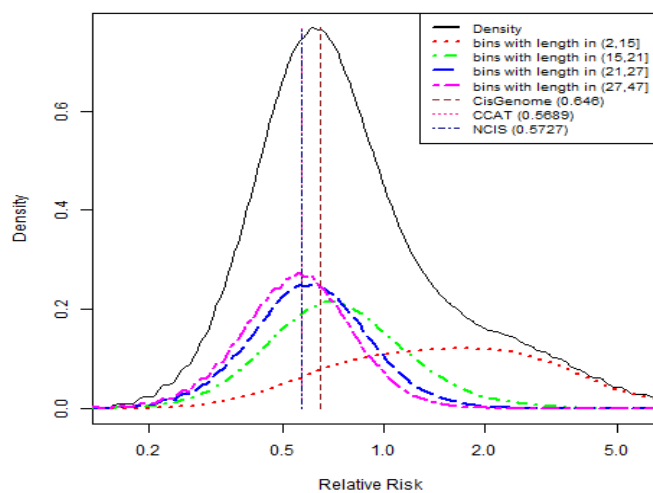

(c) H3K36me3-rep1

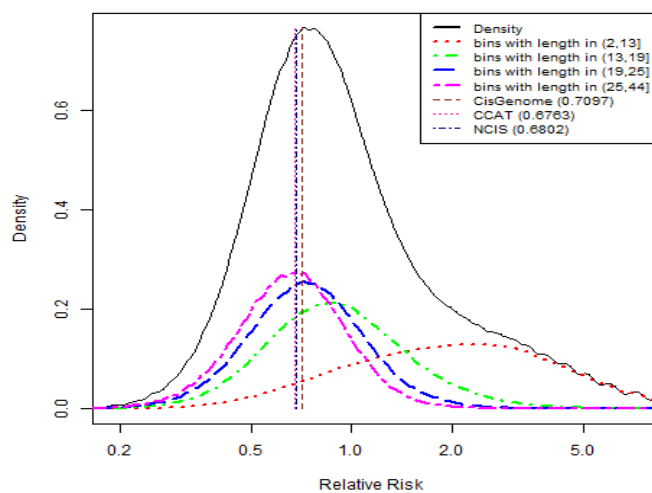

(d) H3K36me3-rep5

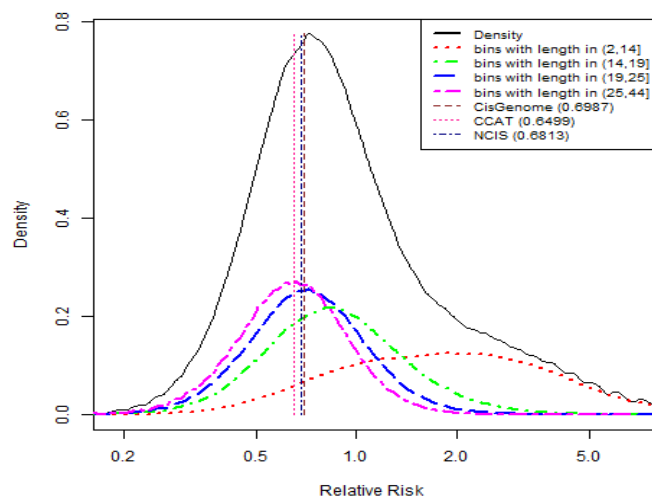

(e) H3K36me3-rep8

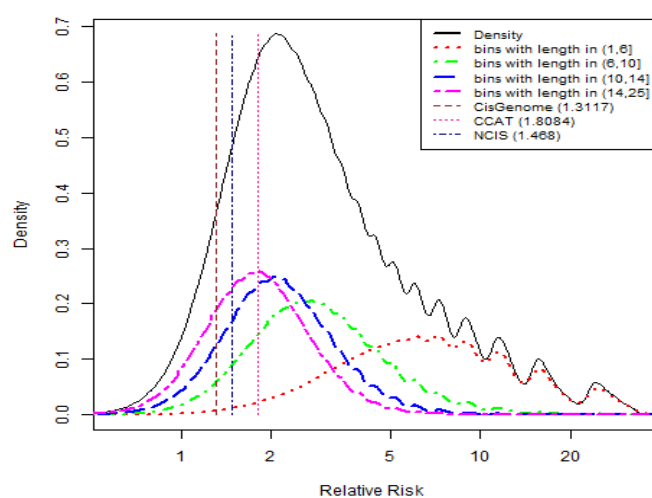

(f) H3K36me3-pooled

Supplement: Additional file 1 — Diagnostic plots. Analogous to Figure 1. Diagnostic plots for six datasets of histone modifications in [38]. The plot refers to K=50. [file 12859_2015_579_MOESM1_ESM.pdf]

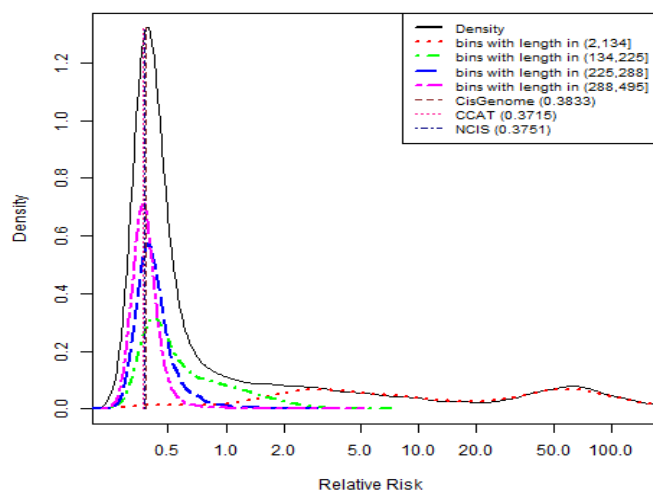

(a) H3K4me3

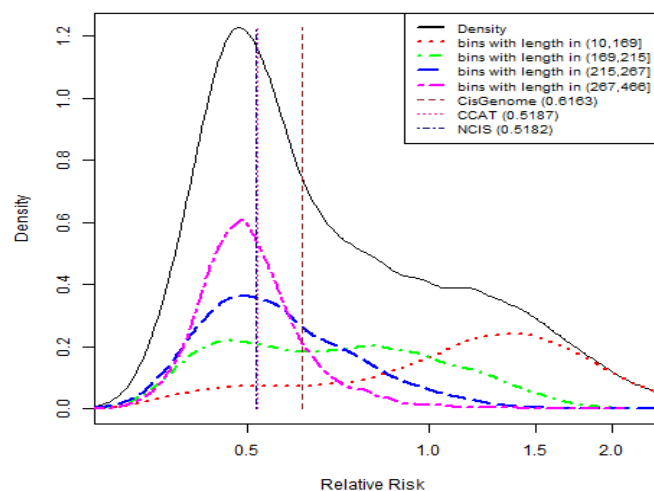

(b) H3K27me3

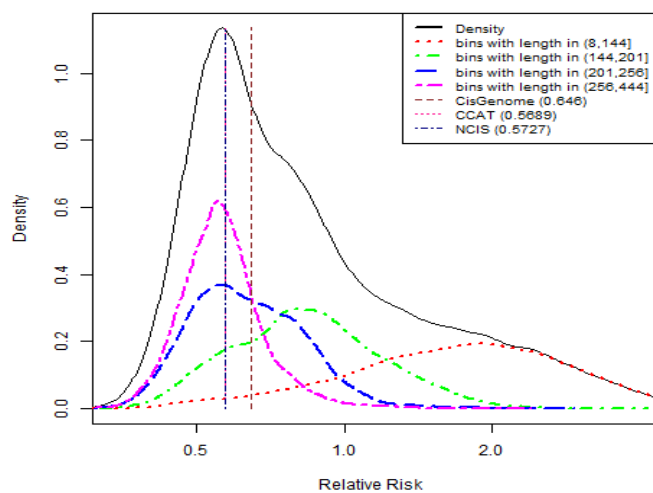

(c) H3K36me3-rep1

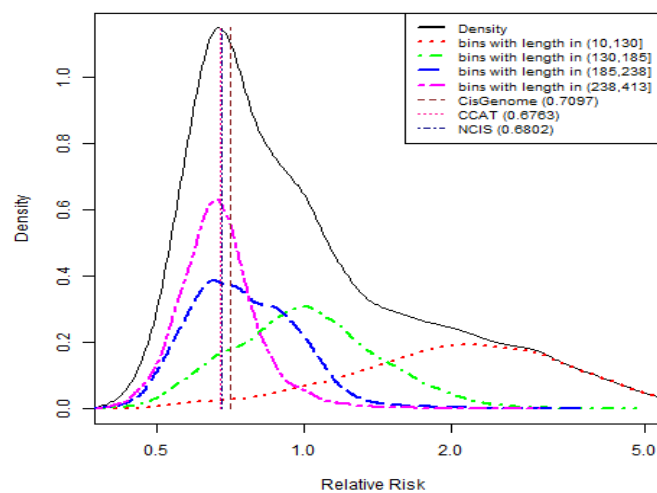

(d) H3K36me3-rep5

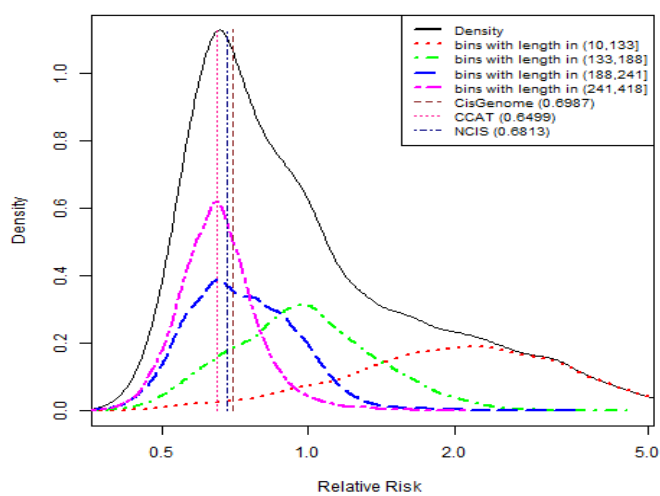

(e) H3K36me3-rep8

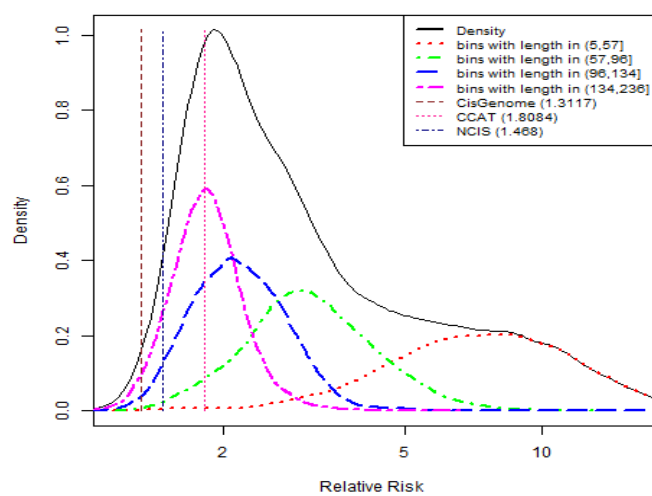

(f) H3K36me3-pooled

Supplement: Additional file 2 — Diagnostic plots. Analogous to Figure 1. Diagnostic plots for six datasets of histone modifications in [38]. The plot refers to K=500. [file 12859_2015_579_MOESM2_ESM.pdf]

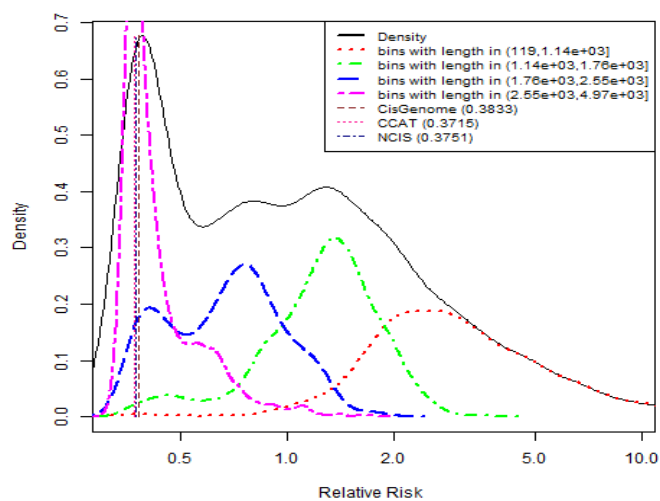

(a) H3K4me3

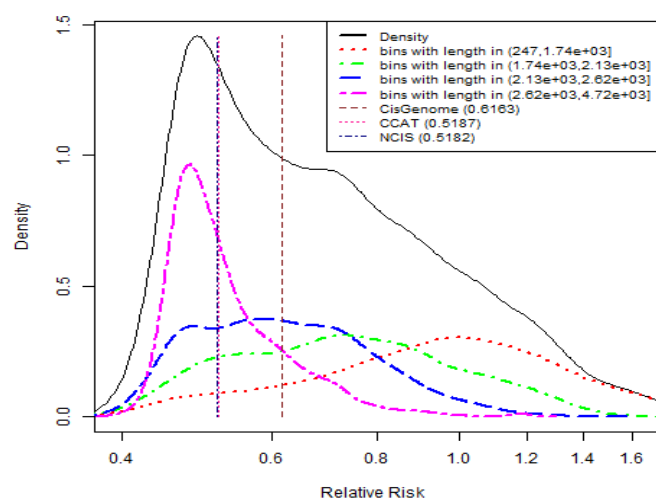

(b) H3K27me3

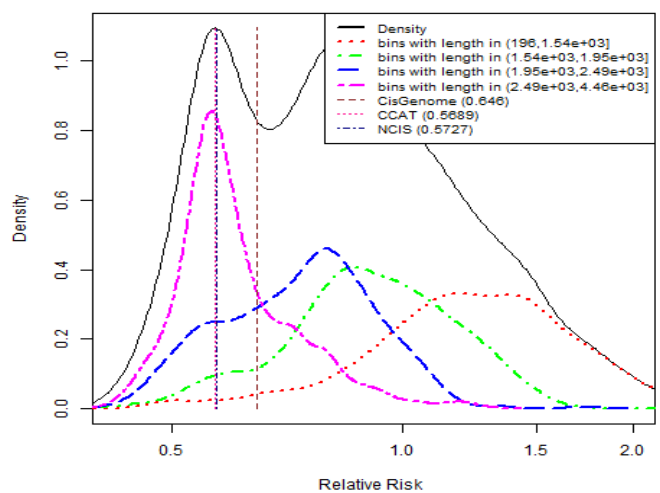

(c) H3K36me3-rep1

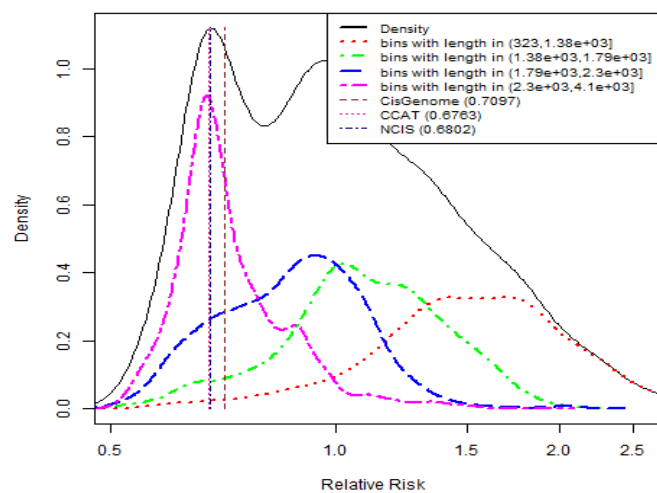

(d) H3K36me3-rep5

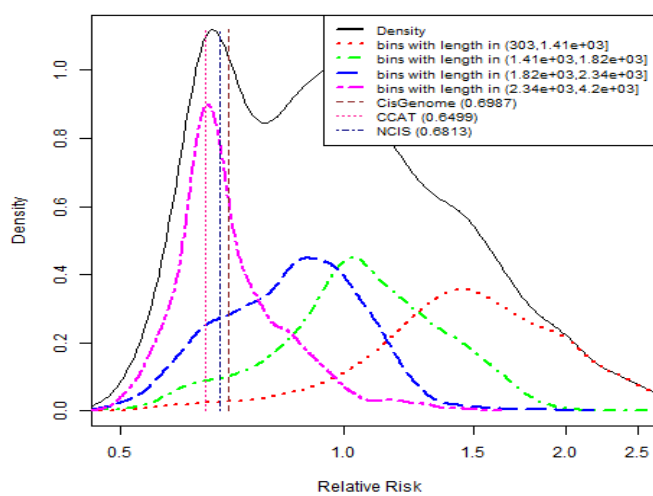

(e) H3K36me3-rep8

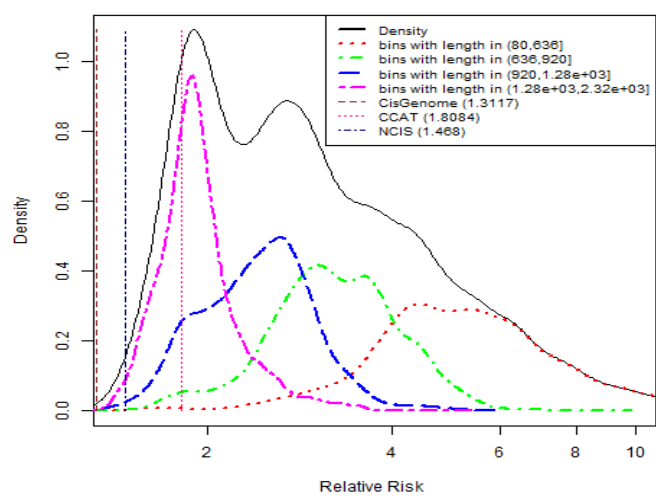

(f) H3K36me3-pooled

Supplement: Additional file 3 — Diagnostic plots. Analogous to Figure 1. Diagnostic plots for six datasets of histone modifications in [38]. The plot refers to K=5000. [file 12859_2015_579_MOESM3_ESM.pdf]

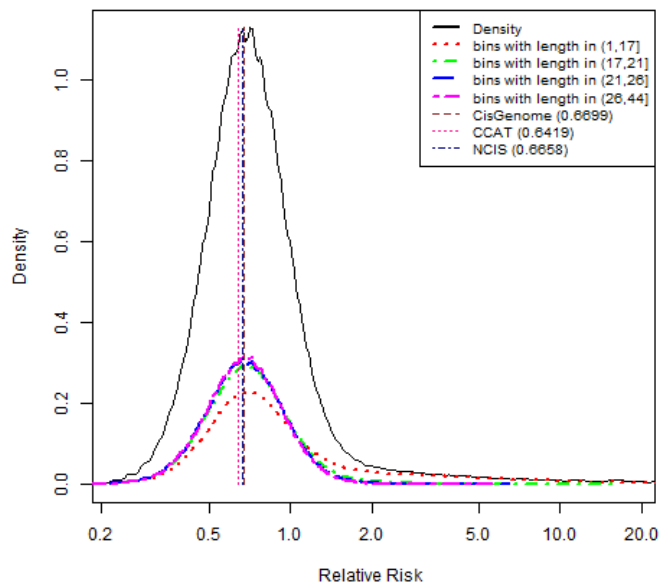

(a) CTCF,  $K = 50$

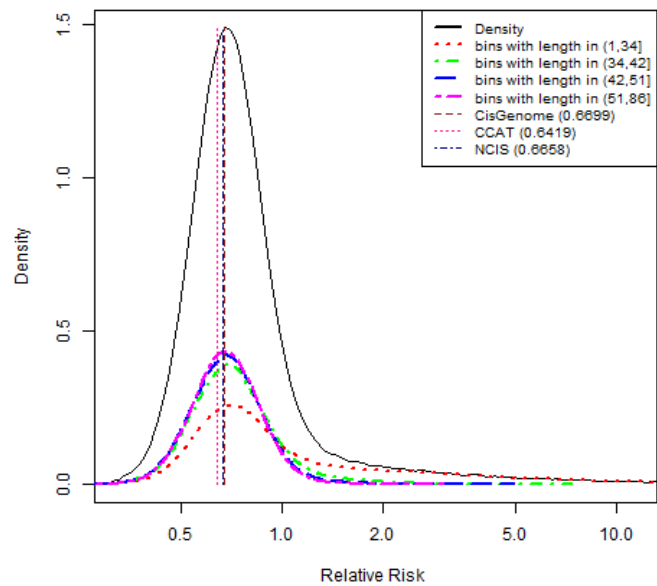

(b) CTCF,  $K = 100$

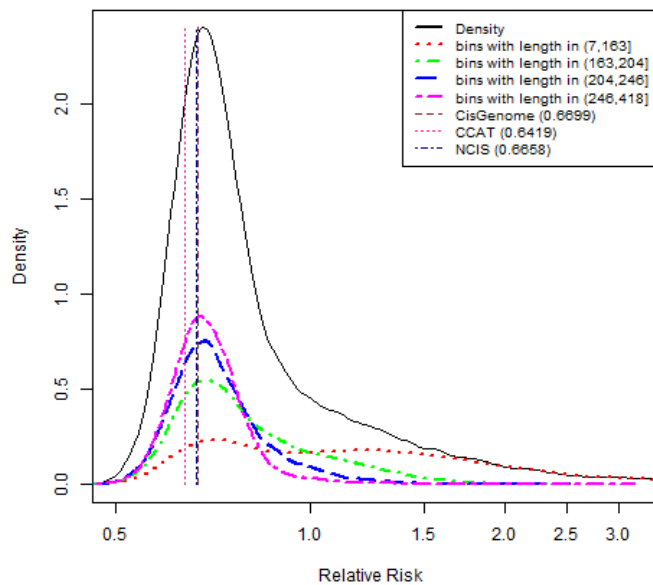

(c) CTCF,  $K = 500$

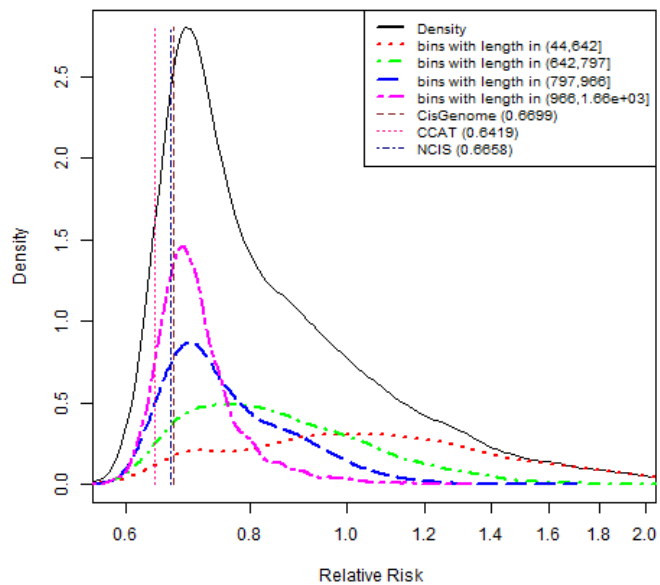

(d) CTCF,  $K = 2000$

Supplement: Additional file 4 — Diagnostic plots. Diagnostic plots for the CTCF dataset in [38]. The plot refers to K=50,100,500 and 2000, respectively. [file 12859_2015_579_MOESM4_ESM.pdf]

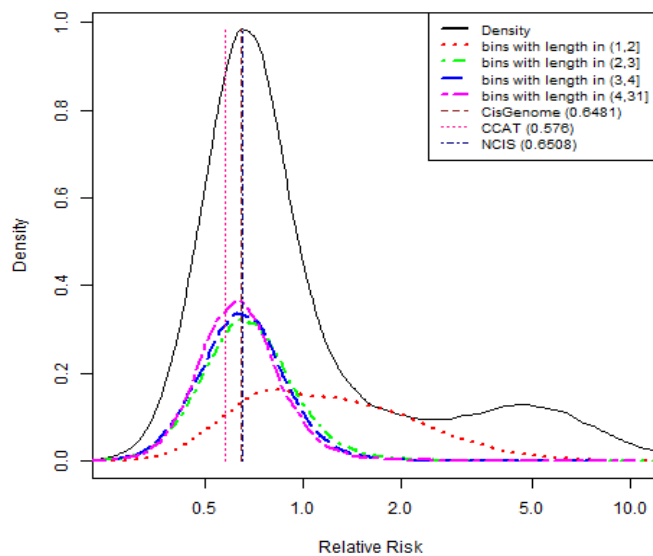

(a) model organism, H3K27me3,  $K = 100$

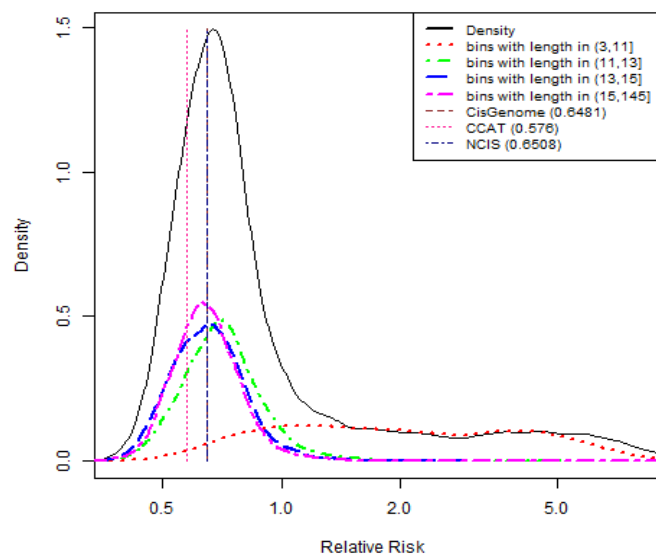

(b) model organism, H3K27me3,  $K = 500$

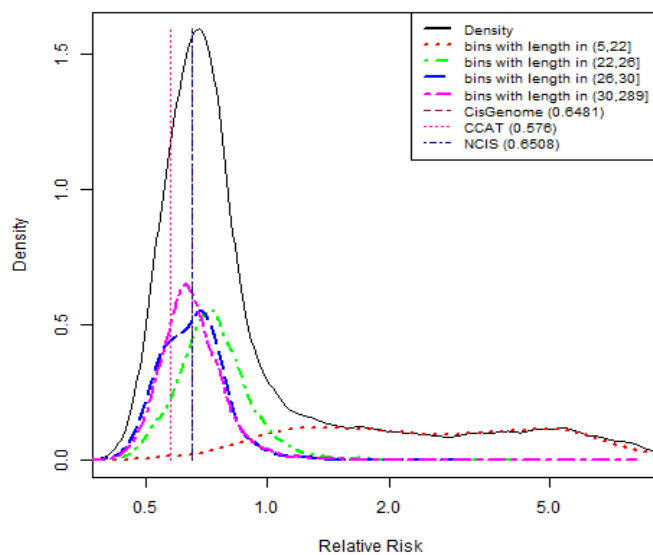

(c) model organism, H3K27me3,  $K = 1000$

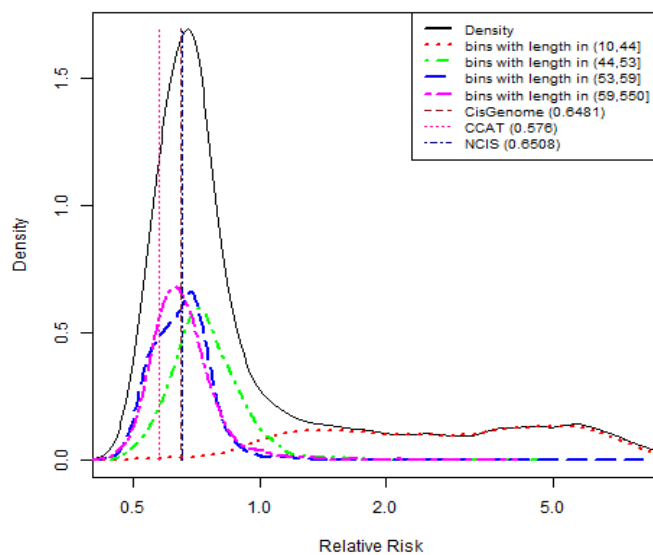

(d) model organism, H3K27me3,  $K = 2000$

Supplement: Additional file 5 — Diagnostic plots. Analogous to Additional file 4. Diagnostic plots for H3K27me3 dataset in modENCODE 3955 (id 1820 vs id 1815). The plot refers to K=100,500,1000 and 2000, respectively. [file 12859_2015_579_MOESM5_ESM.pdf]
