# Supplementary material for: Is this the right normalization? A diagnostic tool for ChIP-seq normalization
Source: BMC Bioinformatics. 2015 May 9;16:150. doi: 10.1186/s12859-015-0579-z (PMC4448883; doi:10.1186/s12859-015-0579-z)
Supplement: Additional file 6 — Proof of Theorem 1. [file 12859_2015_579_MOESM6_ESM.pdf]

Supplementary material for: "Is this the right normalization? A diagnostic tool for ChIP-seq normalization"

Claudia Angelini

Istituto per le Applicazioni del Calcolo, 'Mauro Picone', CNR, Italy

Ruth Heller, Rita Volkinshtein and Daniel Yekutieli,

Department of Statistics and Operations Research, Tel Aviv University, Israel.

Proof of Theorem 1

The proof is based on a very nice recent result of [1]. They defined the following procedure, called SSTP1 in [1], where p_j is the p-value for the jth test sequentially tested out of K potential tests, and $c \in (0,1)$ is a fixed threshold:

1. Find $\hat{k} = \max\left\{k \in K : \frac{1+\#\{j\leq k: p_j > c\}}{\#\{j \leq k: p_j \leq c\} \vee 1} \leq \frac{1-c}{c} q\right\}$, and set $\hat{k} = 0$ if the set is empty.
2. Reject all the hypotheses with $j \leq \hat{k}$ such that $p_j \leq c$.

Their Theorem 3 states that if the null p-values are i.i.d. with p_j stochastically at least as large as $Unif[0,1]$, and independent of the non-nulls, then the FDR of procedure SSTP1 is controlled at the desired level q.

In order to use their result, all we need to do is show that our procedure is a special case of their SSTP1 procedure. To see this, we follow similar steps to their steps in Section 5.2 that shows that the knockoff method is also a special case of SSTP1. Let $G_i = \max\{g(\tilde{N}_{ch}(i), \tilde{N}_{in}(i), r), g(\tilde{N}_{in}(i), \tilde{N}_{ch}(i), 1/r)\}$. Assume without loss of generality that $G_1 \geq \ldots \geq G_B > 0$, where B is the total number of bins considered for enrichment. We set

$$p_i = \begin{cases} 1/2 & \text{if } g(\tilde{N}_{ch}(i), \tilde{N}_{in}(i), r) > g(\tilde{N}_{in}(i), \tilde{N}_{ch}(i), 1/r), \\ 1 & \text{if } g(\tilde{N}_{ch}(i), \tilde{N}_{in}(i), r) < g(\tilde{N}_{in}(i), \tilde{N}_{ch}(i), 1/r). \end{cases}$$

These are valid p-values (i.e., stochastically at least as large as the uniform), since under the assumption of the Theorem,

$$Pr(P_i \leq 1/2) = Pr(P_i = 1/2) \leq 1/2.$$

Let K be the indices of strict inequalities:

$$K = \{k : G_k > G_{k+1}\} \cup B.$$

For $c = 1/2$, the following equalities hold for any $k \in K$:

$$\begin{aligned}
&\frac{1 + \#\{i \in S : g(\tilde{N}_{in}(i), \tilde{N}_{ch}(i), 1/r) \geq G_k\}}{\#\{i \in \mathcal{S}^c : g(\tilde{N}_{ch}(i), \tilde{N}_{in}(i), r) \geq G_k\} \vee 1} \\
&= \frac{1 + \#\{i \leq k, i \in S : g(\tilde{N}_{in}(i), \tilde{N}_{ch}(i), 1/r) \geq G_k\}}{\#\{i \leq k, i \in \mathcal{S}^c : g(\tilde{N}_{ch}(i), \tilde{N}_{in}(i), r) \geq G_k\} \vee 1} \\
&= \frac{1 + \#\{i \leq k, i \in S\}}{\#\{i \leq k, i \in \mathcal{S}^c\} \vee 1} = \frac{1 + \#\{i \leq k : p_i > 1/2\}}{\#\{i \leq k : p_i \leq 1/2\} \vee 1}
\end{aligned}$$

where the first equality follows since the bins are arranged in non-increasing order of G_ks, so for $i > k$ it follows that $G_i < G_k$ and therefore $g(\tilde{N}_{in}(i), \tilde{N}_{ch}(i), 1/r) < G_k$ as well as $g(\tilde{N}_{ch}(i), \tilde{N}_{in}(i), r) < G_k$. The second equality follows as well due to the arrangement, since $i \leq k$ if and only if $G_i \geq G_k$, so we really just count the number of indices in $\mathcal{S} \cap \{1, \ldots, k\}$ in the numerator, and the number of indices in $\mathcal{S}^c \cap \{1, \ldots, k\}$ in the denominator. The last equality follows from the definition of the p_is. Therefore, finding the largest k such that the expression after the last equality is at most q is the same as finding the smallest G_k such that the first expression is at most q. This is equivalent to finding the minimum cut-off threshold T, which is our procedure. Therefore, rejecting the p-values with $p_j \leq 1/2$ is the same as rejecting the bins with enrichment scores below T. The FDR control guarantee in Theorem 3 of [1] thus completes the proof.

References

[1] Barber RF, Candes E: **Controlling the false discovery rate via knockoffs** *arXiv:1404.5609* 2014
