# Supplementary material for: Is this the right normalization? A diagnostic tool for ChIP-seq normalization
Source: BMC Bioinformatics. 2015 May 9;16:150. doi: 10.1186/s12859-015-0579-z (PMC4448883; doi:10.1186/s12859-015-0579-z)
Supplement: Additional file 7 — R function chip _ diagnostics . The supplementary html file illustrates the c h i p_d i a g n o s t i c s function and its usage. [file 12859_2015_579_MOESM7_ESM.zip › 1598762460138458_add7.html]

ChIP-seq normalization diagnostics


# ChIP-seq normalization diagnostics

---

#### Supplementary material of the paper: *“Is this the right normalization? A diagnostic tool for ChIP-seq normalization”*, by C. Angelini, R. Heller, R. Volkinshtein and D. Yekutieli

To generate the diagnostic plot the following standard pre-processing steps are needed: I) Alignment of the ChIP and Input read samples to the reference genome. II) Partitioning of the reference genome into contiguous windows of equal length, and counting of the number of ChIP and Input reads corresponding to each window. To ensure that the bins, constructed by agglomerating the individual windows do not include windows from different chromosomes a variable indicating the window’s chromosome should also be included. The result of the preprocessing, should be a “bindata” object, that is a list of the 4 vectors: (1) Number of ChIP reads in window; (2) Number of Input counts in each window; (3) Window’s chromosome; (4) Window index.

We apply our diagnostics to a bindata object that was constructed from BED files taken from public ChIP-seq data GSM879921 (H3K36me3ChIPSeq-rep5) and GSM879924 (Input control), the genomic locations are according to the mouse genome version 8 (mm8).

The data was pre-processed with read.BED and bin.data functions from the R NCIS package (available as supplementary material of *Liang and Keles, 2012*), using the shift.size=100, zero.filter=FALSE and binsize\_min=200 options. The genome was partitioned in windows of length 200bp, to produce the “bindata” object which serves as input to our diagnostic plot.

```
setwd(mypath)
source("read.BED.R")
source("bin.data.R")

file_chip="GSM879921_3158LAAXX.5-2-removed.bed"
file_input="GSM879924_315BDAAXX.5-2-removed.bed"  

Chip=read.BED(file_chip)

Inp=read.BED(file_input)

binsize=200
shift.size=100

chr.vec=c("chr1", "chr10", "chr11", "chr12", "chr13", "chr14", "chr15", "chr16", "chr17", "chr18", "chr19", "chr2",  "chr3",  "chr4" , "chr5",  "chr6", "chr7",  "chr8", "chr9",  "chrX",  "chrY")

bindata1 <- bin.data(chip.pos=Chip, input.pos=Inp, binsize, shift.size=shift.size, shift.half.size=FALSE, zero.filter=FALSE, by.strand=FALSE, chr.vec=chr.vec, chr.end.max=NULL, by.chr=TRUE)


# reshape the output of ìthe bin.data function 
nchr=length(names(bindata1$chip)) 
chr_list=names(bindata1$chip)
chr_vec=NULL

for (ichr in 1:nchr){
  chr=chr_list[ichr]
  windows_chr=length(bindata1$chip[[chr]])
  temp=rep(chr,windows_chr)
  chr_vec=c(chr_vec, temp)
}

bindata=list(chip_counts=unlist(bindata1$chip, use.names = FALSE), 
             input_counts=unlist(bindata1$input, use.names = FALSE),
             bin_chr=chr_vec)

save("bindata", file="bindataForFigure1d.Rdata")
```

```
str(bindata)
```

```
## List of 3
##  $ chip_counts : int [1:13153645] 0 0 0 0 0 0 0 0 0 0 ...
##  $ input_counts: int [1:13153645] 0 0 0 0 0 0 0 0 0 0 ...
##  $ bin_chr     : chr [1:13153645] "chr1" "chr1" "chr1" "chr1" ...
```

The R function “chip\_diagnostics” generates our diagnostics plot. Its main arguments are the bindata object, the bin total count \(K\) (default = 200), and the estimated normalization factor values \(rhat\\_vect\) and their labels \(rhat\\_legend\). Other optional arguments, \(rhat\\_cols\), \(rhat\\_lwd\), \(dens\\_ltype\), \(dens\\_cols\), and \(dens\\_lwd\), can be used to customize the diagnostic plot in term of line colors, widths and types (default values are given). Furthermore, the two additional parameters \(dens\\_quantiles\) and \(xlim\\_max\) can be used to define the break cut-off and upper-bound of X axis (the given default values are useful in most of the cases).

Note that if several estimates of the normalization factor are available, our function allows examination of all the estimates on the same diagnostic plot.

```
chip_diagnostics  <-  function(bindata, K = 200, rhat_vect, rhat_legend, rhat_ltype=rep(1,length(rhat_vect)), rhat_cols=rep("black",length(rhat_vect)),rhat_lwd=1,dens_ltype=c(1,3,4,5,6), dens_cols=c("black","red","green","blue","magenta"),dens_lwd=2,dens_quantiles=c(0,.25,.5,.75,1),xlim_max=0.99){
  
  #data
  tot.counts   <- bindata$chip_counts + bindata$input_counts
  tot.chip   <- sum(bindata$chip_counts)
  tot.input   <- sum(bindata$input_counts)
  tot.sum   <- sum(tot.counts)
  tot.cumsum    <- cumsum(tot.counts)
  ind.end.chr   <- cumsum(table(bindata$bin_chr))
  
  edec.tot.count.num   <- K
  
  #  2. Create EDEC bins
  
  brks.vec          <- sort(unique(c(tot.cumsum[ind.end.chr],seq(1,tot.sum,by=edec.tot.count.num))))
  cut.vec               <- cut(tot.cumsum,brks.vec)
  
  edec.input            <- sapply(split(bindata$input_counts,cut.vec),sum)
  edec.chip         <- sapply(split(bindata$chip_counts,cut.vec),sum)
  edec.length           <- sapply(split(bindata$chip_counts,cut.vec),length)
  edec.tot          <- edec.input + edec.chip
  
  #  2.a Find subset of "good" bin  
  plt.ind   <- (edec.tot.count.num*0.95 < edec.tot)  & (edec.tot  < quantile(edec.tot,xlim_max)) &
    (quantile(edec.length,0.01) < edec.length) &  (edec.length < quantile(edec.length,xlim_max))
  
  edec.input        <- edec.input[plt.ind]
  edec.chip     <- edec.chip[plt.ind]
  edec.length       <- edec.length[plt.ind]
  edec.tot      <- edec.tot[plt.ind]
  
  
  # 2.b Parition "good" bins into acording to bin length into 4 sparsity classes according to 
  # the length of the bin (= number of windows in bin)  short bins are dense and long bins are sparse:
  #  QQ4 is 4th quartile of longest and sparsest bins
  
  cut.vec               <- cut(edec.length,quantile(edec.length,prob=dens_quantiles))
  edec.input.QQ4        <- split(edec.input,cut.vec)[[4]]
  edec.chip.QQ4     <- split(edec.chip,cut.vec)[[4]]
  
  
  #  3. Draw logit densities for all "good" bins
  
  log.RR <- log(edec.chip / edec.input)
  dens.tot  <- density(log.RR)
  dens.QQ   <- lapply(split(log.RR,cut.vec),density)
  
  
  #************************ Diagnostics Plot
  
  plot(exp(dens.tot$x), dens.tot$y,type="l",xlim=exp(quantile(log.RR,prob=c(0.001,xlim_max))),main="",ylab = "Density",xlab="Relative Risk ", lty=dens_ltype[1],col = dens_cols[1], log = "x")
  lines(exp(dens.QQ[[1]]$x),dens.QQ[[1]]$y/4,lty=dens_ltype[2], col=dens_cols[2],lwd=dens_lwd)
  lines(exp(dens.QQ[[2]]$x),dens.QQ[[2]]$y/4,lty=dens_ltype[3], col=dens_cols[3],lwd=dens_lwd)
  lines(exp(dens.QQ[[3]]$x),dens.QQ[[3]]$y/4,lty=dens_ltype[4], col=dens_cols[4],lwd=dens_lwd)
  lines(exp(dens.QQ[[4]]$x),dens.QQ[[4]]$y/4,lty=dens_ltype[5], col=dens_cols[5],lwd=dens_lwd)
  
  logit.pi.ch  <- (rhat_vect)
  
  for (i in seq(1:length(rhat_vect))){
    lines(logit.pi.ch[i],max(dens.tot$y),type='h',lty=rhat_ltype[i],col=rhat_cols[i],lwd=rhat_lwd) 
  }
  
  legend("topright",legend=c("Density",paste("bins with length in ",levels(cut.vec),sep=""),
                             paste(rhat_legend," (",rhat_vect,")",sep="")),lty=c(dens_ltype,rhat_ltype), 
         col=c(dens_cols,rhat_cols), 
         lwd=c(rep(dens_lwd,5),rep(1,length(rhat_vect))), cex=0.85) 
  
  #************************  
}
```

We generate a diagnostics plot with K = 200 for Figure 1(d) of the manuscript.

```
chip_diagnostics(bindata, K=200 , rhat_vect=c(0.7097,0.6802,0.6763), rhat_legend=c("CisGenome","CCAT","NCIS"),rhat_ltype=c(2,3,4),rhat_cols=c("brown4","deeppink","navy"))
```
